# Environmental DNA (eDNA) reveals potential for interoceanic fish invasions across the Panama Canal

**DOI:** 10.1002/ece3.9675

**Published:** 2023-01-29

**Authors:** Lennart Schreiber, Gustavo A. Castellanos‐Galindo, D. Ross Robertson, Mark Torchin, Karina Chavarria, Silke Laakmann, Kristin Saltonstall

**Affiliations:** ^1^ Smithsonian Tropical Research Institute Balboa Panama; ^2^ Faculty of Biology & Chemistry University of Bremen Bremen Germany; ^3^ Leibniz Institute of Freshwater Ecology and Inland Fisheries (IGB) Berlin Germany; ^4^ Helmholtz Institute for Functional Marine Biodiversity at the University of Oldenburg (HIFMB) Oldenburg Germany; ^5^ Alfred‐Wegener‐Institute, Helmholtz Centre for Polar and Marine Research Bremerhaven Germany

**Keywords:** COI metabarcoding, environmental DNA (eDNA), fish invasion, gillnet fish sampling, Panama Canal

## Abstract

Interoceanic canals can facilitate biological invasions as they connect the world's oceans and remove dispersal barriers between bioregions. As a consequence, multiple opportunities for biotic exchange arise and the resulting establishment of migrant species often causes adverse ecological and economic impacts. The Panama Canal is a key region for biotic exchange as it connects the Pacific and Atlantic Oceans in Central America. In this study, we used two complementary methods (environmental DNA (eDNA) metabarcoding and gillnetting) to survey fish communities in this unique waterway. Using COI (cytochrome oxidase subunit I) metabarcoding, we detected a total of 142 fish species, including evidence for the presence of sixteen Atlantic and eight Pacific marine fish in different freshwater sections of the Canal. Of these, nine are potentially new records. Molecular data did not capture all species caught with gillnets, but generally provided a more complete image of the known fish fauna as more small‐bodied fish species were detected. Diversity indices based on eDNA surveys revealed significant differences across different sections of the Canal reflecting in part the prevailing environmental conditions. The observed increase in the presence of marine fish species in the Canal indicates a growing potential for interoceanic fish invasions. The potential ecological and evolutionary consequences of this increase in marine fishes are not only restricted to the fish fauna in the Canal as they could also impact adjacent ecosystems in the Pacific and Atlantic Oceans.

## Graphical abstract

The Panama Canal is a key region for biotic exchange as it connects the Pacific and Atlantic Oceans in Central America. In this study, we used two complementary methods (environmental DNA (eDNA) metabarcoding and gillnetting) to survey fish communities in this unique waterway. The observed increase in the presence of marine fish species in the Canal indicates a growing potential for interoceanic fish invasions across the Isthmus.

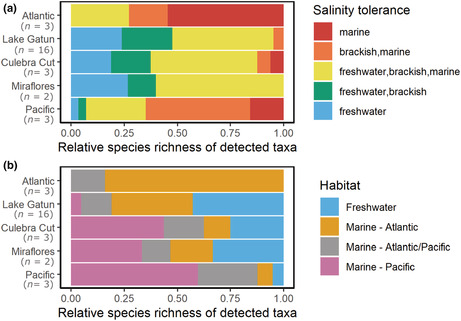

## 1 INTRODUCTION

Physical and biological barriers define the limits of different habitats and thus determine species distributions and evolutionary processes. Biotic interchange occurs when these barriers disappear, allowing species to disperse into new habitats with previously distinct biota. In this context, anthropogenic changes such as the construction of canals have the potential to remove barriers and have been shown to facilitate biological invasions across multiple spatial scales (Gollasch et al., 2006). The resulting changes in species composition have implications for the ecological processes driving evolution, and can ultimately lead to the extinction of local species or the emergence of novel taxa via speciation (Vermeij, 1991).

In geological time scales, the Central American Isthmus has been a key region for biotic exchange as it connects the landmasses of North and South America and has served as a bridge for plants and animals to move between these two continents. However, the rise of the isthmus also created a barrier between the Atlantic and Pacific Oceans, leading to diversification of their respective marine biotas (Lessios, 2008). This physical barrier, which has existed for millions of years (Coates & Stallard, 2013; Montes et al., 2015; O'Dea et al., 2016), was altered by the construction of the Panama Canal about 100 years ago. The Canal, which is often referred to as the most important maritime gateway of the Western Hemisphere (Manfredo, 1993), is crucial in shortening distances for global maritime transportation, but can also serve as a potential passageway for marine species between the two oceans (Ruiz et al., 2009).

The main shipping channel of the Panama Canal lies 26 m above sea level and the lock systems at either entrance are gravity fed, thus freshwater flows from the Canal into the locks and out into the ocean, limiting salt water incursion (Jongeling et al., 2008). This design feature has historically been an important factor in limiting or reducing species passage through the Canal, as salinity levels in the Canal are low and most marine species cannot tolerate them (Cohen, 2006 and references herein; Hildebrand, 1939). However, Lake Gatun, which is a large artificial freshwater lake forming much of the Canal, is known to experience migrations of euryhaline species through the locks (Hildebrand, 1939; McCosker & Dawson, 1975; Sharpe et al., 2017). The salinity in some parts of the Canal undergoes seasonal changes caused by varying precipitation, evaporation and shipping intensity (Salgado et al., 2020). The recent expansion of the Canal and installation of a new set of larger locks on either end using a different gate system with water conservation basins has raised concerns about salt water incursion into the waterway and the potential for more marine species to disperse between the oceans (Castellanos‐Galindo et al., 2020; Hewitt et al., 2006; Muirhead et al., 2015).

The fish fauna of the Canal has undergone several changes since it was constructed (Castellanos‐Galindo et al., 2020; Hildebrand, 1939; Rubinoff & Rubinoff, 1968; Sharpe et al., 2017; Zaret & Paine, 1973). Initially, two evolutionary distinct native freshwater communities from either side of the continental divide (Rio Grande on the Pacific side and Rio Chagres on the Atlantic side) were connected when Lake Gatun was created in 1913 (Meek & Hildebrand, 1916; Smith et al., 2004). Soon after the opening of the Canal, marine species were encountered in the locks during maintenance works and the first evidence of a successful transit from ocean to ocean emerged when the Atlantic Tarpon (*Megalops atlanticus*) was recorded in the Miraflores Locks on the Pacific side of the Isthmus by Samuel Hildebrand in 1937 (Hildebrand, 1939). Since then, at least 16 migrant fish species have been reported in different sections of the Canal but only four species are known to have successfully invaded and established in the opposite ocean basin (Cohen, 2006). However, the occurrence of other non‐native organisms at the Atlantic/Pacific entrances of the Canal (most likely mediated by shipping) has been documented (e.g. the bivalve *Anomia peruviana* (Schlöder et al., 2013), the crustacean *Rhithropanopeus harrisii* (Roche et al., 2009) or for an overview see Torchin et al., 2021). Both the ability to detect species prior to successful establishment and observations of species movements that do not result in invasions are crucial for understanding and managing biological invasions (Morisette et al., 2021). Therefore, efficient tools for monitoring are needed to detect first signs/occurrences of non‐native species. In recent years, environmental DNA (eDNA) metabarcoding has been shown to be a promising method for detecting fish species in aquatic ecosystems such as canals (McDevitt et al., 2019), rivers (Pont et al., 2018), lakes (Jerde et al., 2011; Valdez‐Moreno et al., 2019) and the ocean (Thomsen et al., 2012; Valdivia‐Carrillo et al., 2021). Both intra‐ and extra‐organismal DNA can be extracted from water samples (Barnes & Turner, 2016) and its persistence can range from days to weeks under freshwater conditions (Dejean et al., 2011; Pilliod et al., 2014). The distribution of eDNA can vary across space based on currents, boat activity, and proximity to moving water, such as streams and rivers, giving it a larger spatial footprint than classical aquatic monitoring techniques, such as gillnetting (Harrison et al., 2019; Pont et al., 2018). Furthermore, eDNA‐based surveys are particularly suited for the detection of rare and cryptic species, which may also be non‐native (e.g. Thomsen et al., 2012) and may be overlooked by traditional surveys.

In this study, we combine eDNA metabarcoding and gillnetting surveys to investigate the presence and distribution of marine fish species across the Panama Canal after the recent expansion of this shipping corridor. Since critical components of the Panama Canal (e.g. the locks) are only rarely accessible and large parts of Lake Gatun are characterized by a complex shape and extensive shallow areas (Zaret, 1984), eDNA‐based surveys allowed us to sample across the entire Canal as water sampling can be performed without the constraints typically associated with fish sampling involving nets. We collected and processed water samples from sites spanning the length of the Canal and also from the Pacific and Atlantic entrances, on the seaward sides of the locks. This is the first time eDNA has been used to survey fishes in the Panama Canal and may serve as a baseline for future assessments. The objectives were to: (1) characterize the fish community of the Panama Canal, with a focus on Lake Gatun, using eDNA metabarcoding; (2) compare the fish diversity of a subset of sites in the lake determined by traditional surveys (gillnets) with eDNA analyses; and (3) identify the presence of invading marine fish species along the entire length of the Canal.

## 2 MATERIALS AND METHODS

### 2.1 Study site

**FIGURE 1:**
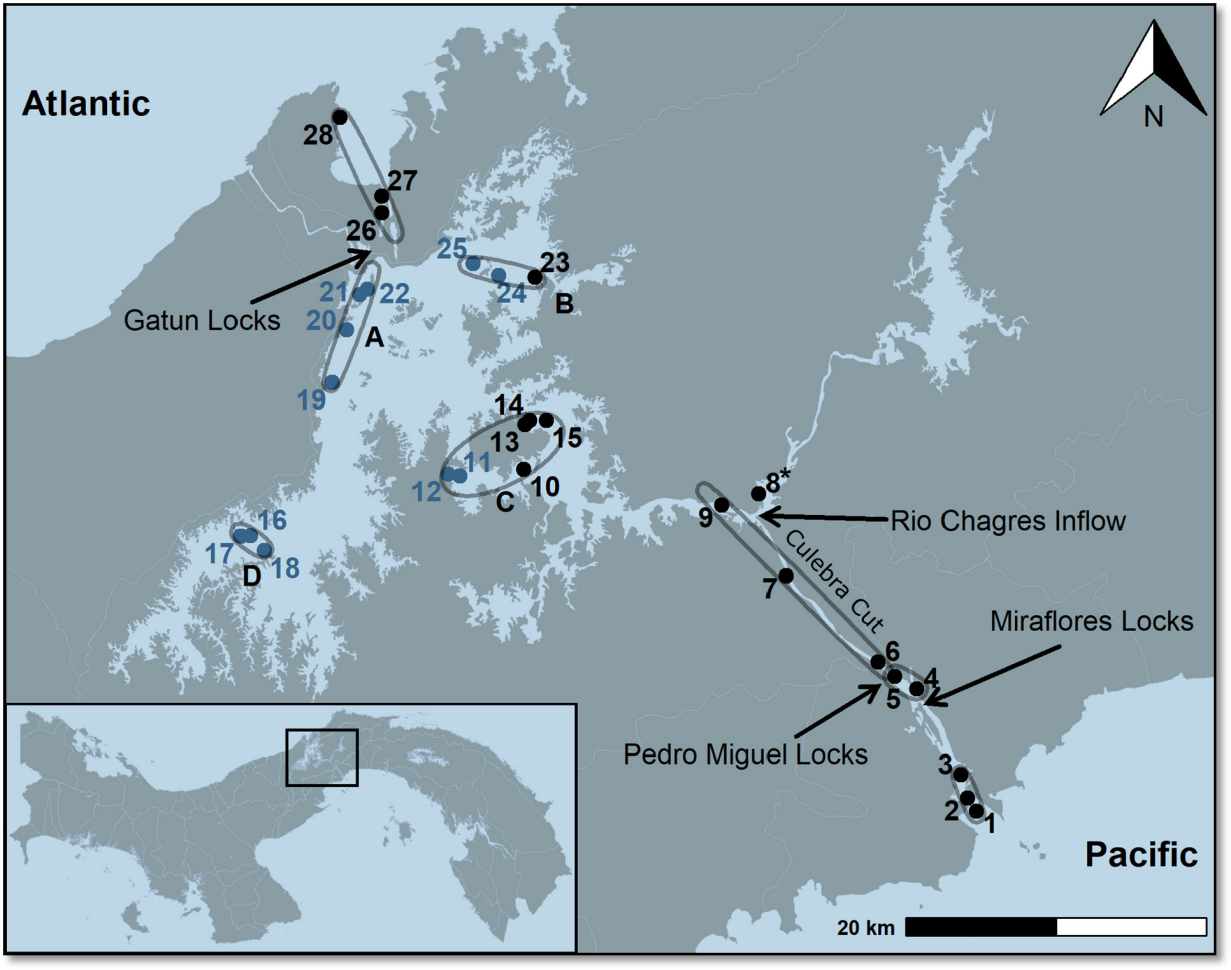
Map of the Panama Canal with key locations (black arrows) and subsections (gray spheres) indicated. Black numbers indicate sampling sites where only eDNA surveys were conducted (*n* = 18), whereas blue numbers mark sites surveyed using both gillnets and eDNA (*n* = 11). Sampling sites were grouped into sections of the canal: Pacific (1, 2, 3), Miraflores (4, 5), Culebra cut (6, 7, 9), Lake Gatun (10–25) and Atlantic (26, 27, 28). Fish that were detected at site #8 (Rio Chagres) were not included in the main analysis as this site is located outside of the canal. Letters A – D indicate subsections of Lake Gatun.

The Panama Canal is a ~ 82 km long artificial waterbody that was completed in 1914 and bridges the continental divide in Central America, connecting the Atlantic with the Pacific Ocean (Figure 1). Each year ~13,000 vessels cross the Canal, thus making it one of the most important waterways in the world (ACP, 2020). In crossing from the Atlantic to the Pacific, vessels first enter a series of three locks which lift them to the level of Lake Gatun. This lake was formed by Gatun Dam and is supplemented by water flowing down the Chagres River from Lake Alajuela. Lake Gatun has a maximum depth of 30 m, lies 26 m above sea level and covers an area of approximately 425 km^2^ (Zaret, 1984). The shipping channel through the lake varies in depth from 13.6–30 m and extends for about 37 km to Gamboa where the Chagres River joins the Lake and the Culebra Cut begins. The Culebra Cut, which is an excavation through the continental divide, extends for about 13 km to Pedro Miguel Locks, the first set of three locks on the Pacific side of the Canal. These locks lower vessels 9 m to Miraflores Lake, where they pass through a 1.2 km channel to the final two‐stepped locks at Miraflores where they are lowered to sea level (Figure 1). Between 2007 and 2016, the operational capacity of the Canal was expanded and a new set of larger‐capacity locks was installed on either end which use a different gate system and water conservation basins which recycle water. On the Pacific side, the new locks also bypass Miraflores Lake, opening directly into the Culebra Cut. The predicted effects of the expansion on the salinity of the Canal are twofold: the larger locks may allow more salt water to enter Lake Gatun from the oceans, and the lock water may be less diluted with fresh water from Lake Gatun as the water is being reused through water‐saving basins during operation (Wijsman, 2013).

For our purposes, we divided the Canal into sections which correspond to the major artificial and natural barriers: Atlantic, Lake Gatun, Culebra Cut, Miraflores, and Pacific (Figure 1). While the locks are the main physical barriers to interoceanic species dispersal, the inflow of the Chagres river near where the Culebra Cut merges with Lake Gatun (Gamboa) also acts as a possible barrier by lowering the salinity through the introduction of freshwater, although the flow of this river is controlled by an upstream dam and varies depending on the seasonality of rainfall.

### 2.2 eDNA sample collection

Six one‐liter replicate water samples were collected from each of the 28 sites (*n* = 168) between November 2019 and February 2020 (Table S1 and Figure 1). The sites were accessed either by boat, when sampling was conducted from the vessel (*n* = 19 sites), or by foot, when the sampling was conducted from the shore (*n* = 9 sites) due to security regulations preventing sampling from a vessel. Water was collected from approximately 20 cm below the surface using sterile 1 L Nalgene® bottles mounted on a custom‐built 1 m extension stick. At sites where gillnets were employed, eDNA samples were collected just prior to net retrieval. Immediately after collection, bottles were placed in clean Ziploc® bags and stored at 4°C until filtration. During each sampling event, a sterile 1 L Nalgene® bottle filled with distilled water from the lab was left open during sample collection to check for contamination and serve as a field blank (*n* = 9, multiple sites were sampled during one sampling event). Salinity and temperature were measured at approximately 20 cm depth at each sampling site using a handheld YSI™ multi‐parameter instrument (YSI, Yellow Springs, OH, USA).

Water samples were vacuum‐filtered at the Naos Marine Laboratories (Smithsonian Tropical Research Institute, Panama) within 24 hours of collection using MF‐Millipore™ mixed cellulose membrane filters with 0.45 μm pore size. In addition to the field blanks, lab blanks (*n* = 8) of 1 L of Milli‐Q purified water were filtered and subsequently processed in the same way as the samples. Filters were stored dry at −20°C in 5 ml sterile low‐bind tubes until extraction. Sampling bottles were washed, soaked in bleach overnight and autoclaved before reuse and handled with gloves to minimize contamination.

### 2.3 DNA extraction, library preparation and sequencing

In preparation for DNA extraction, filters were cut up into small pieces using sterile forceps and scissors to improve DNA yield. Forceps and scissors were bleached, immersed in ethanol and flame sterilized between filters. Four of the six water sample replicates from each site were extracted using the DNeasy® PowerWater® extraction kit (Qiagen) with the following modifications to the manufacturer's protocol: After adding PW1 reagent, tubes were briefly vortexed and then incubated at 55 °C for 10 min to increase the DNA yield. Subsequently, tubes were vortexed for 8 min. For the remaining protocol, tubes were always centrifuged at 10,000 *g* instead of 13,000 *g*. In the final step, 60 μl of EB solution was added to increase the concentration of the extracted DNA. Reagent blanks were also extracted to control for contamination during extractions (*n* = 11).

After each batch of extractions, we estimated the DNA concentration (ng/μl) and measured the 260/230 and 260/280 ratios using a NanoDrop ND‐100 spectrophotometer (ThermoFisher Scientific) (see Table S2). For some samples, we encountered low DNA concentrations as well as 260/280 ratios that pointed towards low levels of extraction efficiency. We thus decided to extract the remaining two replicates from each site using a 2% CTAB protocol (Doyle & Doyle, 1987). This non‐commercial extraction method has recently received increased attention in eDNA studies due to its high extraction efficiency, low per sample cost and robustness in the presence of inhibitors (Geerts et al., 2018; Hunter et al., 2019; Turner et al., 2014).

We used a two‐stage polymerase chain reaction (PCR) protocol to amplify a fragment of the cytochrome c oxidase I (COI) region and construct our sequencing library (detailed information on PCR conditions are compiled in Table S3). PCR1 used fish‐specific primers (AquaF2/C_FishR1 & AquaF3/C_FishR1 (Ivanova et al., 2007; Valdez‐Moreno et al., 2019) modified to include partial Illumina sequencing adapters on their 5′ ends, Table S3) to amplify a 184–187 bp fragment. We chose to target the COI locus as it provides the highest availability of reference barcodes (89% coverage for fish species previously found in the Canal, based on surveying BOLD entries on 1st March 2022), which directly determines the ability to identify the generated sequences. PCR plates always included one negative control well (containing 2 μl sterile H_2_O) and one positive control well (containing 1 μl of fish tissue DNA extract ‐ *Chaetoton humeralis* and *Paranthias colonus* and 1 μl H_2_O). Since these two species are reef fish from the Pacific with little tolerance of low salinities, their occurrence in the Canal is very unlikely, thus making them ideal for tracking possible contamination during lab work. PCR products were run on agarose gels to check if amplification was successful. Overall, more samples showed visible bands when amplified with the primer combination AquaF3/C_FishR1 (hereafter F3), even though multiple bands were observed in some cases (thus indicating the presence of non‐specific amplification). Only few samples showed bands when amplified with AquaF2/C_FishR1 (hereafter F2), so we decided to continue with PCR2 for all samples amplified with F3 and only for the subset of samples that showed visible bands with F2. All indexed PCR2 products were pooled to make a library which was then run on an agarose gel. The band of the targeted size was cut and cleaned with the Qiagen MinElute Gel Purification kit (#28606). Subsequently, the library was checked on an Agilent BioAnalyzer and its concentration was quantified using a Qubit fluorometer. Finally, the library was sequenced on the Illumina MiSeq sequencing platform using a 2 × 250 bp PE Reagent kit. DNA extraction, amplification and sequencing were carried out at the Smithsonian Tropical Research Institute (STRI) Naos Molecular Lab in Panama.

### 2.4 Gill net surveys

To test whether eDNA‐based species identifications differed from traditional sampling methods and validate detections of non‐native fish species, we deployed gillnets at a subset of sampling sites (Table S1 and Figure 1), building on historical sampling efforts by Zaret and Paine (1973) and more recently by Sharpe et al. (2017). The gillnets, which were 45 m long, 3 m high and consist of six segments with mesh sizes ranging from 1 to 6 inches [e.g. 2.54–15.24 cm], were placed in the shallow littoral zones of Lake Gatun. In total, eleven sites were sampled in November 2019 (Figure 1). At seven sites, nets were set in the evening and retrieved early in the morning of the next day. At the remaining four sites, nets were set around midday and retrieved after 2 h (Table S1). All collections were approved by Panama's Ministry of Environment (Permit # SC/A‐36‐2019) and STRI's Institutional Animal Care and Use Committee (Protocol # 2018–0415‐2021‐A4).

### 2.5 Data analyses

All data analyses were performed in R (R Core Team, 2020). Cutadapt v1.15 (Martin, 2011) and DADA2 v. 1.14.0 (Callahan et al., 2016, 2017) were used to remove primer sequences, quality‐filter reads (filterAndTrim with maxN = 0, maxEE = c(2, 4), truncQ = 2), infer exact amplicon sequence variants (ASVs), merge paired reads and remove chimeric sequences (removeBimeraDenovo, method = “consensus”). Sequences within the size range of 100–205 bp were retained and the taxonomy of the remaining 7400 ASVs was assigned (minimum similarity >97%) using BOLD's integrated alignment tool (Ratnasingham & Hebert, 2007). In some cases, barcodes from multiple congeners matched the submitted sequences. This may be caused by a lack of taxonomic resolution in the short fragments that we amplified or by human‐derived errors manifested in the reference database. Fortunately, BOLD allows tagging of misidentifications so barcodes with questionable status were excluded from the results. Matches were also checked against Eschmeyer's Catalog of Fishes (Fricke et al., 2020) and names adjusted when necessary to reflect their current valid taxonomic status. Finally, information about the geographic range and salinity tolerances for all detected species were compared with the species lists from the Smithsonian Tropical Research Institute Caribbean/Eastern Pacific shore fish databases (Robertson & Allen, 2015; Robertson & van Tassell, 2015, retrieved 31/05/2020) to identify non‐native and potentially invasive fish species. Ambiguous ID's were discussed with experts of the local fish fauna (Angulo, A., González Gutiérrez, R., Robertson, D. R. and Victor, B., personal communication, May 28, 2020).

**FIGURE 2:**
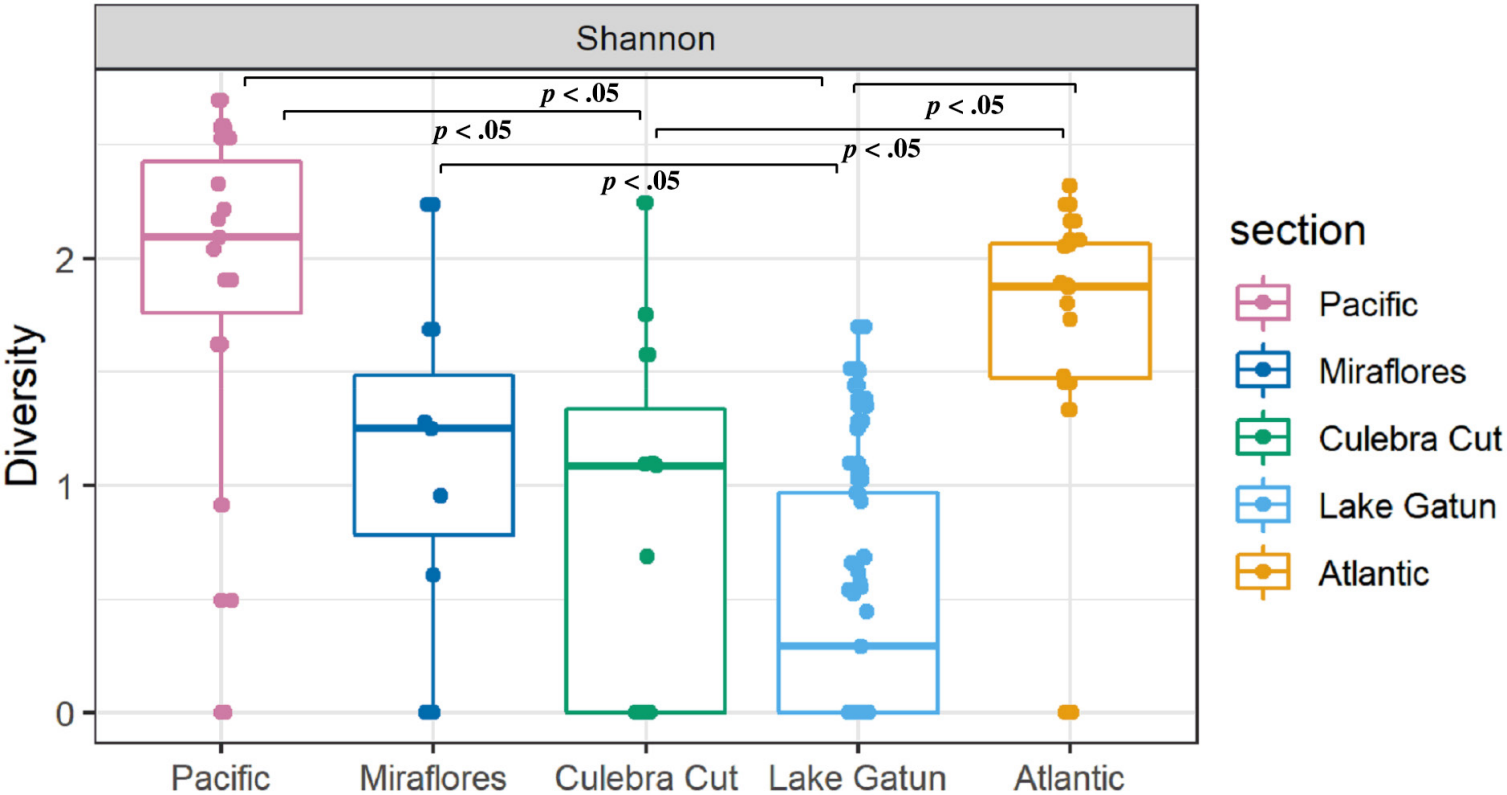
Shannon diversity of fish communities in different sections of the Panama Canal detected with COI metabarcoding using primer combination F3/C_FishR1. Each point represents an eDNA sample. Significant pairwise‐comparisons (Tukey's HSD) are indicated with *p* < .05.

**FIGURE 3:**
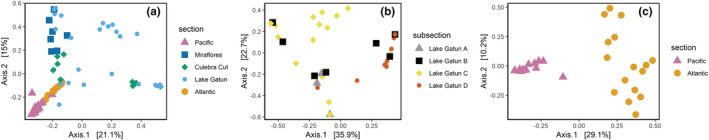
MDS ordinations (bray–Curtis dissimilarity index) of fish communities across different sections of the Panama Canal detected with COI metabarcoding: (a) entire canal, (b) subsections of Lake Gatun, and (c) Pacific and Atlantic entrances. Each point on the plots represents an eDNA sample. The Pacific and Atlantic sections are outside of the lock systems on either end of the canal (sea level); Miraflores is a lake located between the two sets of locks on the Pacific side of the canal (16 m asl); Culebra cut and Gatun Lake (26 m asl) contain the main shipping channel. The subsections of Lake Gatun vary in salinity: A (site No: 19–22; mean salinity: 0.45 ppt) is more influenced by salt water incursions due to its proximity to the Atlantic locks than B (site No: 23–25; mean salinity: 0.21 ppt), C (site No: 10–15; mean salinity: 0.26 ppt) and D (site No: 16–18; mean salinity: 0.19 ppt). Axis labels indicate the percent variation explained by the axis.

**FIGURE 4:**
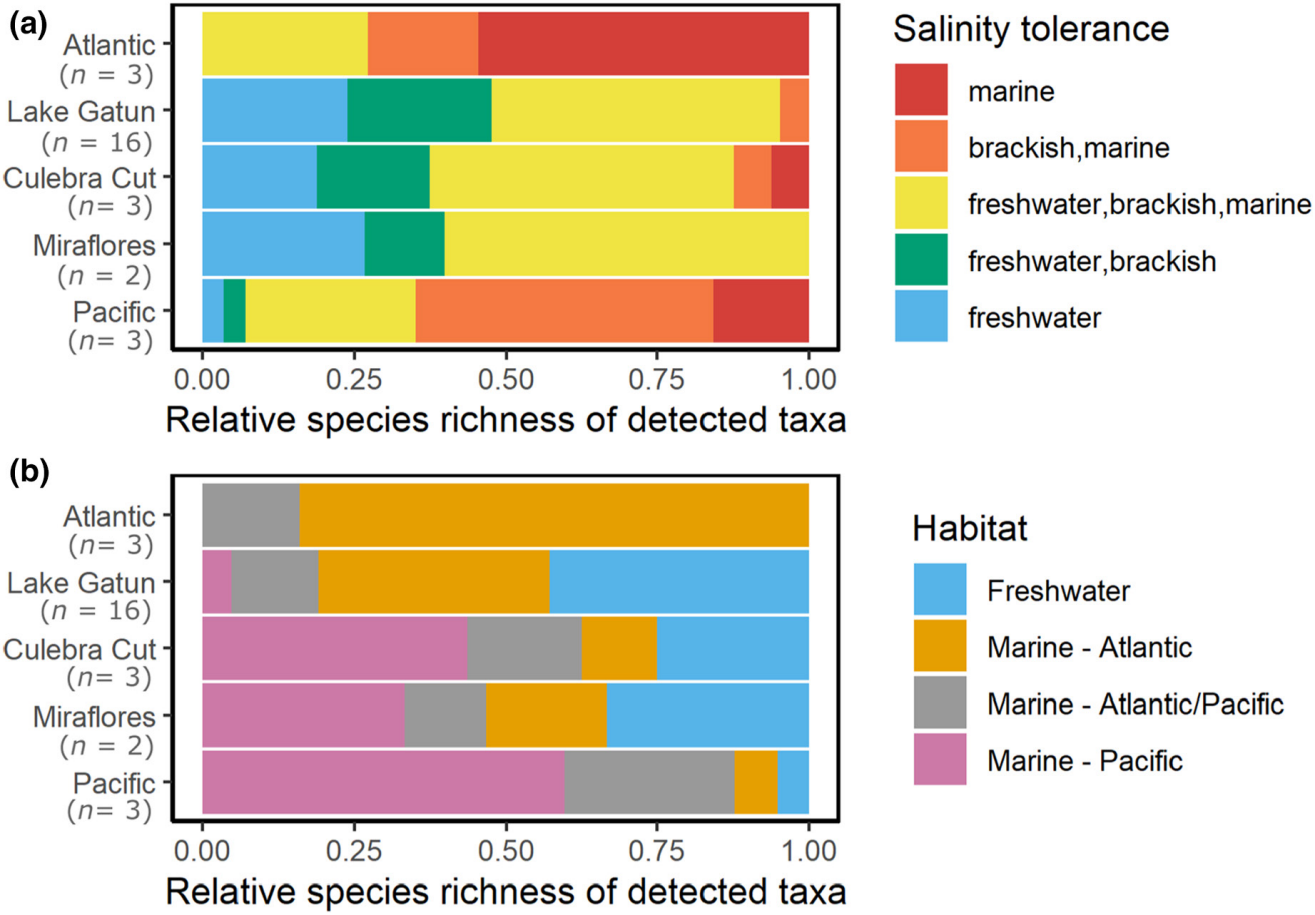
Relative species richness of fish species detected using eDNA (primer F3) across different sections of the Panama Canal classified by habitat (a) and salinity tolerance (b). Habitat classifications were assigned using biogeodb.stri.si.edu/caribbean and biogeodb.stri.si.edu/sftep. Genus level identifications of ambiguous marine origin are classified as Pacific/Atlantic and marked in gray. The number of sampling sites in each section are indicated in parentheses. The total number of fish species detected in each section: Atlantic = 53, Lake Gatun = 23, Culebra cut = 21, Miraflores = 21 and Pacific = 63.

**FIGURE 5:**
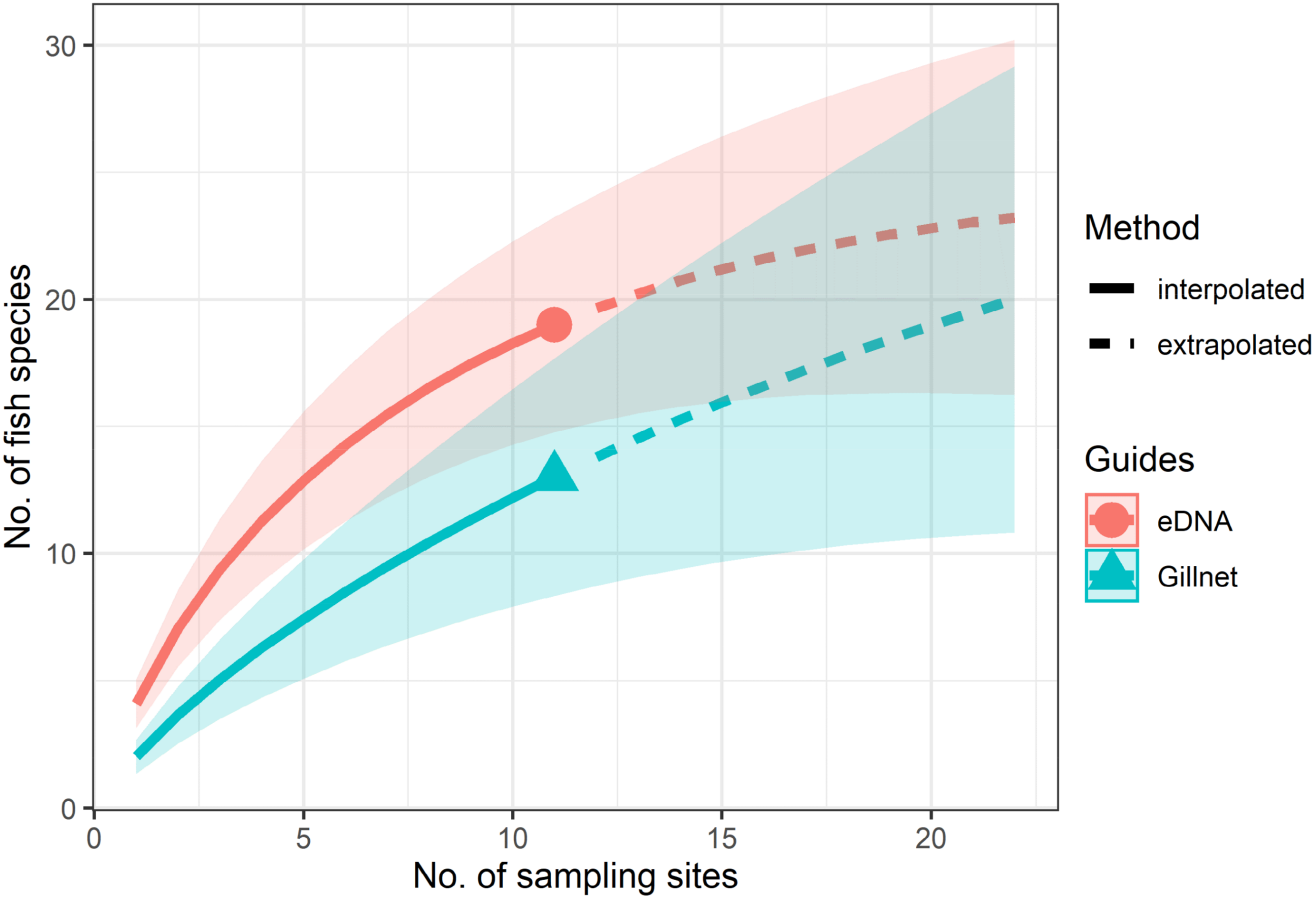
Numbers of fish species detected at 11 sampling sites in Lake Gatun using eDNA and gillnet surveys. Shaded areas represent 95% confidence intervals.

Prior to further analysis, only reads taxonomically classified as fish were retained and a minimum threshold of 10 reads per ASV was implemented to reduce the probability of considering artefactual sequences. All ASV's assigned to the same species were then merged and blanks were examined for signs of contamination. Only two reagent blanks showed contamination out of 29 blanks sequenced. One blank showed reads assigned to *P. colonus* (positive control) and one showed reads assigned to *Anchoa* sp. We also detected reads matching positive control species (*C. humeralis* and *P. colonus*) in a total of seven field samples. Contamination with *C. humeralis* and *P.colonus* DNA is most likely linked to our use of undiluted tissue extracts as positive controls during preparation of PCR1, thus introducing a high risk of contamination early in the laboratory workflow. We excluded all reads matching the positive control species when running our main analysis as we are confident that the reads derived from the positive control do not represent any real occurrences of the two species at our study sites. Reads matching *Anchoa* sp. may indicate cross‐contamination which could have occurred at different stages of the field or lab work (Schnell et al., 2015) and a recent study published by Bohnmann et al., 2021 suggests that the untagged 2‐step indexing approach used in this study has a higher risk of cross‐contamination between PCR products (Bohmann et al., 2021). In addition, due to the fact that all samples were run with the F3 primer combination, but only 50% with both primer sets (F2 and F3), care was taken to separate data for the subsequent analysis. When looking at the general ability of eDNA metabarcoding to detect fish in one section of the Canal, data from both primers was used (Table 1 and Table S5). However, when comparing detections between sites, only data generated with F3 were used (Figures 2, 3, 4, 5) to prevent the introduction of methodological bias, where some samples are overrepresented by two PCR reactions.

**TABLE 1:** The recorded fish fauna of the Panama Canal. References in brackets correspond to: 1944 = Breder, 1944; 1973 = Zaret & Paine, 1973; 2004 = Averza Colamarco et al., 2004; 2017 = Sharpe et al., 2017; 2020 = Castellanos‐Galindo et al., 2020. Introduced species are marked in bold and species marked with (**!**) are potential new records or species for the study area. Predicted habitats (f = freshwater, b = brackish, m = marine) and geographic ranges (A = Atlantic, P = Pacific; As = Atlantic slope, Ps = Pacific slope) were retrieved from biogeodb.stri.si.edu/caribbean and biogeodb.stri.si.edu/sftep and iucnredlist.org (individual species assessments retrieved 10/06/2020). Species originating from the Atlantic and Pacific are marked with color (orange and purple, respectively) to allow for easier identification. Detection indicates method (gillnet or eDNA) and F2/F3 stands for the primer set used to detect the corresponding species. Availability of reference COI barcodes for each species is also indicated.

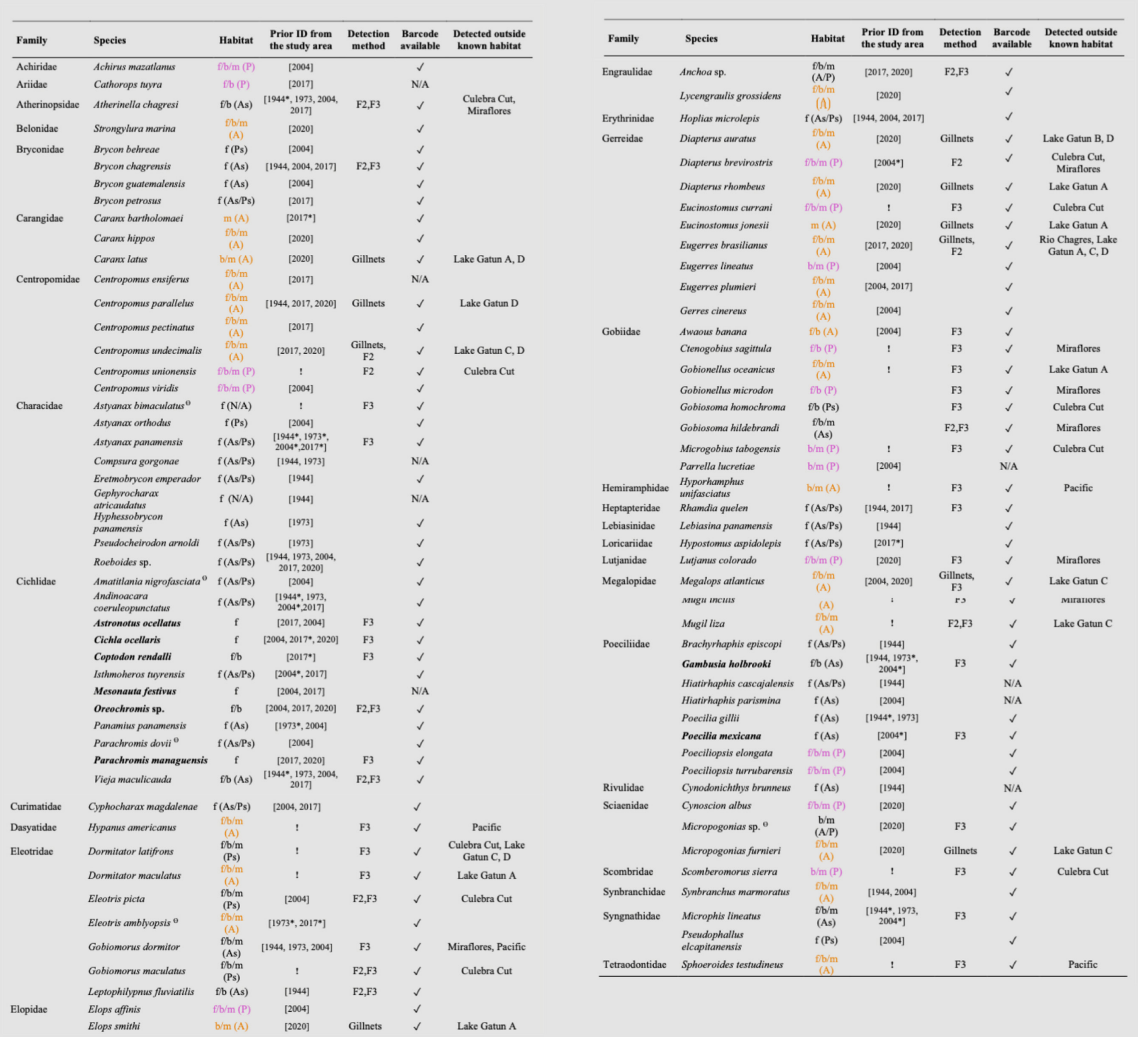

*Note*: Ɵ = Ambiguous identifications, as discussed in the supplemental material; **!** = Potential new record or species for the study area; * = Change in nomenclature, following Eschmeyer's Catalog of Fishes; F2/F3 = The primer set which produced the respective sequences; ✓ = Species with DNA barcode present in the BOLD dataset; N/A = No barcode sequence available.

The five most recent traditional sampling campaigns in Lake Gatun (Averza Colamarco et al., 2004; Breder, 1944; Castellanos‐Galindo et al., 2020; Sharpe et al., 2017; Zaret & Paine, 1973) were used to construct a baseline list of fish species known to occur inside the freshwater segment of the Panama Canal. All species detected (eDNA & gillnet sampling) were then added to the list and information about barcode coverage was retrieved from BOLD (Table 1). In order to compare detection success from eDNA and gillnet surveys, species accumulation curves were computed using the *iNext* R package (Hsieh et al., 2016).

To assess differences in fish diversity across sections of the Canal (i.e. Atlantic, Lake Gatun, Culebra Cut, Miraflores and Pacific), we used a combination of visualization and statistical tools, mostly from the R package *vegan* v2.5‐6 (Oksanen et al., 2019). All samples that did not contain any sequences identified as fish (zero read samples) were removed and sequence reads were Hellinger‐transformed. In this transformation, the data are normalized by taking the square root of the relative abundance of sequencing reads to avoid biases caused by large differences in the number of reads retrieved for the detected species (Laporte et al., 2021; Legendre & Gallagher, 2001). Rarefaction curves of all samples (prior to any transformation) showing the range of sequencing depth across the samples demonstrated that taxonomic diversity was captured in most samples with as few as 500 reads (Figure S1). We then calculated alpha‐diversity indices (Shannon, Inverse Simpson) for each sample and compared different sections of the Canal using ANOVA, after verifying normality of these data using Shapiro–Wilk tests. Finally, Tukey's HSD post‐hoc tests were run to determine which pairwise comparisons were different.

Beta‐diversity was explored using Bray–Curtis dissimilarity indices ordinated for all samples using Multidimensional Scaling (MDS). Proximity of sampling sites as well as measured salinities were used to group Lake Gatun sites into four subsections: Lake Gatun A (sites 19–22), Lake Gatun B (sites 23–25), Lake Gatun C (sites 10–15) and Lake Gatun D (sites 16–18; Figure 1). Before differences between sections were tested for significance, beta dispersion values were calculated to test for homogeneity of variances. The subsequent pair‐wise permutation test showed that at least some sections have different dispersions and we refrained from running a PERMANOVA (Anderson, 2017) for the full dataset and instead used ANOSIM, which does not assume equal group variances (Clarke, 1993). Since variances between subsections of Lake Gatun proved to be homogenous, a PERMANOVA and subsequent pairwise tests were performed on these data.

## 3 RESULTS

### 3.1 Salinity measurements

Surface water salinities measured in this study fell into three categories, defined as freshwater (<0.5 parts per thousand (ppt)), brackish (0.5–24 ppt) and marine (coastal with strong freshwater influence: 24–40 ppt) (adapted after IAL and IUBS, 1958). Both the Pacific and the Atlantic entrances to the Canal showed salinities typical for coastal marine environments influenced by freshwater runoff, with values ranging from 24.5–30.1 ppt. The water of Miraflores Lake (Cocodrilos #5) was brackish with a salinity of 1.2 ppt. All sites inside Lake Gatun and Culebra Cut showed values below 0.5 ppt and thus were classified as freshwater. Salinities varied depending on proximity to the locks and riverine freshwater inflow. Sites near the Gatun locks reached salinities close to 0.5 ppt (e.g. Isla Guarapo (#22): 0.48 ppt). When moving away from the Gatun locks, salinities generally decreased, but remained above 0.25 ppt near the shipping channel in the middle of the Canal (e.g. BCI3 (#14): 0.27 ppt). On the Pacific side, the salinity was also higher close to the locks (Paraiso (#6): 0.31 ppt), but quickly decreased when approaching the Chagres River inflow (Culebra Cut (#7): 0.08 ppt; Figure 1, Table S1).

### 3.2 Overview of sequencing data

If not otherwise indicated, the following results describe sequence data generated with both primer sets (F2 and F3). A total of 179,335 sequencing reads remained after processing the raw reads, excluding all non‐fish reads and imposing the minimum read threshold (see Table S4 for details on the number of reads retained at each step of the bioinformatics workflow). Fourteen of our 168 samples did not contain fish sequences (~8.3%) and the maximum number of fish species detected in one sample was 17 (a replicate collected at Puente Americas (#2)). The mean number of species detected per sample was 2.9 for data generated with primer set F3 and 1.6 for data generated with primer set F2. In some cases, detected sequences could only be identified to family or genus level. For data generated with F3, reads assigned to *Anchoa* sp. and *Atherinella chagresi* together made up ~45% of all fish reads, followed by *Cetengraulis mysticetus* (12.2%), *Strongylura exilis* (6.8%) and *Dormitator latifrons* (5.0%). In contrast, for data generated with F2, 20.5% of fish reads were assigned to *Anchoa* sp., followed by *Oreochromis* sp. (19.3%), *Diapterus brevirostris* (16.5%), *Eleotris picta* (14.4%) and *Brycon chagrensis* (7.2%). However, not all samples amplified with F2, so the detection efficiency and composition of species could not be directly compared.

#### 3.2.1 Species distribution patterns across the Panama Canal

In total, 127 unique species were detected across our study site using primer set F3. Marine sites had significantly higher diversity than freshwater sites (ANOVA Shannon: *F*_4, 93_ = 18.1, *p* < .005; Inverse Simpson: *F*_4, 93_ = 21.0, *p* < .005, Figure 2, Table S6), with 63 species in the Pacific section (3 sampling sites) and 53 species in the Atlantic (3 sites). Culebra Cut and Miraflores had the fewest species (*n* = 21), with similar sampling effort (3 and 2 sites). Lake Gatun had intermediate numbers of species (*n* = 23), even though our sampling effort was the highest (16 sites). Community diversity of eDNA samples from different sections of the Canal revealed some degree of spatial grouping, but also suggests interchange between the fish communities (Figure 3). Samples from the Pacific and Atlantic clustered together, as marine species were detected across samples from different sites (particularly *Anchoa* sp.), whereas many freshwater species were only detected in individual samples. The majority of samples from Miraflores and Culebra Cut clustered with the Pacific and Atlantic samples along MDS axis 1 but separated along axis 2, while samples from Lake Gatun were scattered across both axes 1 and 2 (Figure 3a). When ordinated alone, the subsections of Lake Gatun were not very clearly separated (Figure 3b) but pair‐wise comparisons showed community differences that were statistically significant (*p* < .05) for all pairs except Lake Gatun B x Lake Gatun D which have similar salinity levels (Table S1).When ordinated separately, the Pacific and Atlantic communities are clearly divided (Figure 3c; PERMANOVA: *p* < .001, *R* ^2^ = .27), reflecting their biogeographic distinctiveness. ANOSIM revealed that fish communities differed significantly between the five Canal sections (Bray‐Curtis: *p* = .001, *R* ^2^ = .40).

#### 3.2.2 Community composition and potential candidates for interoceanic establishment.

Using both eDNA metabarcoding and gillnetting, a total of eight Pacific and 16 Atlantic fish species were detected inside the Canal (Table 1). Many of these were found at sampling sites closest to the locks (four Atlantic species in Lake Gatun subsections A/B and all eight Pacific species in Culebra Cut/Miraflores Lake), but we also detected marine species in portions of the Canal that are more distant from their source ocean (e.g. eight Atlantic species in Lake Gatun subsections C/D, one Atlantic species in Culebra Cut and one Atlantic species in Miraflores).

Most of the species that we detected (74%) have a broad salinity tolerance classified as brackish (and freshwater/marine), 16% only tolerate marine conditions, and 10% of species are purely freshwater (Figure 4a). Less than 25% of species detected at the Pacific sites outside the locks are classified as purely marine and we found two species at those sites that are classified as purely freshwater (*Brycon chagrensis* and *Rhamdia quelen*). In contrast, more than 50% of species recorded outside the Atlantic entrance are classified as purely marine. Overall, Lake Gatun and Culebra Cut had the highest relative species richness of freshwater taxa, but only 25% of their communities are classified as pure freshwater species and the majority of the detected species are known to also tolerate brackish or even marine conditions (Figure 4a). In total, eDNA metabarcoding detected 37 taxa in the freshwater part of the Canal (Lake Gatun and Culebra Cut), of which 32 were identified to species and five to genus level. Of these 37 taxa, only 14 are native freshwater/brackish species, seven are introduced freshwater/brackish species (e.g. *Cichla ocellaris*), and the remaining 16 are marine fish species (e.g. *Megalops atlanticus*), including nine potentially new marine records for the uninterrupted waterbody of Lake Gatun and Culebra Cut (e.g. the Atlantic species *Gobionellus oceanicus* and the two Pacific species *Centropomus unionensis* and *Scomberomorus sierra*, also see Table 1). We also confirm the continued presence of five historically reported interoceanic migrants in different sections of the Canal (Table 1): *Eleotris picta* (Pezold & Cage, 2002), *Gobiosoma homochroma* and *Gobiosoma hildebrandi* (Hildebrand, 1939; McCosker & Dawson, 1975)*, Megalops atlanticus* (Castellanos‐Galindo et al., 2019 and references therein; Hildebrand, 1937) and *Microphis lineatus* (McCosker & Dawson, 1975). Furthermore, we detected the genetic material of three other marine species which had previously been recorded in the Canal: *Centropomus undecimalis* (Sharpe et al., 2017)*, Diapterus brevirostris* (Averza Colamarco et al., 2004) and *Eugerres brasilianus* (Sharpe et al., 2017). Thus far, interoceanic migration has not been reported for these three species, but their continued presence in the Canal makes them potential candidates. In addition, we found sequences from three Atlantic species (*Sphoeroides testudineus, Hypanus americanus* and *Hyporhamphus unifasciatus*) outside the Pacific entrance of the Canal (Table 1), suggesting that these species may have successfully crossed the Canal. However, detections were limited to a small number of samples (*S. testudineus* and *H. unifasciatus* in one and *H. americanus* in two of 18 Pacific samples) and we did not detect these species inside the Canal or on the Atlantic side. The genetic material of another four species whose native ranges include both the Pacific and the Atlantic (*Diodon holocanthus, Mugil hospes, Awaous banana* and *Dajaus monticola*) was also detected (Table S5). In some cases, the detected sequences could only be assigned to genus level (*n* = 17) and, as these genera are represented by species both in the Pacific and the Atlantic, their associated source habitat could not be clearly determined (Figure 4b).

#### 3.2.3 Comparison of eDNA and gillnet survey methods in Lake Gatun

We caught 35 individuals from 13 fish species at the eleven gillnet sampling sites. Most individuals were medium‐ (10–20 cm) to large‐bodied (>20 cm) and belong to seven families, of which Gerreidae (four species), Cichlidae (three species) and Centropomidae (two species) were the most representative. However, eDNA metabarcoding from the gillnet sampling sites only detected 19 fish species, as generated with the F3 primer set. The two survey techniques also showed contrasting results, with only two records overlapping at the species level, and another two at the genus level. Fifteen species were detected using eDNA but not with gillnets, and nine species caught with gillnets were not detected using eDNA, even though COI barcodes are available for these species. When the number of unique species detected was plotted against the number of sampling sites, eDNA surveys revealed higher species diversity than gillnets suggesting that more species are detected with eDNA given a similar sampling effort (Figure 5).

## 4 DISCUSSION

The Panama Canal, with its defined boundaries and historically well‐documented fish community is an ideal site to test the efficacy of eDNA methods and predict potential for interoceanic fish invasions. Our results support previous observations that fish communities along the Panama Canal are changing, with more and larger marine fishes reported inside the Canal (Castellanos‐Galindo et al., 2020). This could amplify the effects that earlier intentional introductions, such as the introduction of the peacock bass to Lake Gatun (Sharpe et al., 2017; Zaret & Paine, 1973), have had on the native freshwater community. Perhaps more importantly, the increased presence of marine fishes in the Panama Canal can potentially serve as a steppingstone for interoceanic invasions and subsequently impact native biodiversity in both the Caribbean and Tropical Eastern Pacific.

### 4.1 Changes in the fish community of Lake Gatun and Culebra cut

Over a period of 76 years, a total of 78 fish species have been recorded in Lake Gatun and the Culebra Cut using traditional survey techniques such as gillnets and beach seines (Table 1). Historically, studies have found that the fish community of Lake Gatun primarily consisted of native and some introduced freshwater species (Averza Colamarco et al., 2004; Breder, 1944; Sharpe et al., 2017; Zaret & Paine, 1973). Our results are consistent with the more recent study of Castellanos‐Galindo et al. (2020) indicating an increase in the presence of marine fishes in the freshwater sections of the Panama Canal (Table 1). Specifically, 16 out of 37 fishes (43%) detected with eDNA and 17 out of 21 species (81%) caught with gillnets in Lake Gatun are marine fishes native to either the Pacific or Atlantic oceans. Of these, nine of the species detected with eDNA are potential new records for Lake Gatun/Culebra Cut (Table 1). Only 25% of the species detected in the freshwater segment of the Canal were classified freshwater species and species with brackish tolerance were dominant. In contrast, only 10 out of 32 species (31%) caught during the last comprehensive gillnet sampling campaign in 2014–2016 were marine (Sharpe et al., 2017). Furthermore, we did not detect many of the small‐bodied (<10 cm) freshwater species, which had previously been recorded at Lake Gatun (e.g. *Mesonauta festivus*, *Brycon petrosus* and *Andinoacara coeruleopunctatus* (Sharpe et al., 2017) or *Panamius panamensis* and *Amatitlania nigrofasciata* (Averza Colamarco et al., 2004)). Unlike previous studies (Sharpe et al., 2017; Zaret & Paine, 1973), we rarely encountered the peacock bass *Cichla ocellaris*, in either our gillnet (only 2 specimens at one site) and eDNA surveys (a total of 112 sequence reads at three sites). This freshwater predator, which was first introduced to Panama in 1969, is thought to have had a strong impact on the structure of the Canal's fish community by preying on small native fishes (Sharpe et al., 2017; Zaret & Paine, 1973). Our findings also corroborate anecdotal information from recreational fishers, which document an increase in the presence of large marine fish inside the Canal since 2016 (Castellanos‐Galindo et al., 2020).

The observed community shift, with the presence of multiple large marine predatory fishes (e.g. Jacks and Atlantic Tarpon) and a decreased prevalence of native and non‐native freshwater fishes, may be attributed to changing salinity conditions. Long‐term salinity measurements, that provide a good representation of the spatial and seasonal variation in this parameter, are needed to understand how salinity influences marine fish from entering, crossing and/or persisting in the Canal. Further ecological consequences of this community shift may be the reduction of species diversity or extirpation of native freshwater fishes of the scale documented in the 1970 s after the introduction of the peacock bass (Sharpe et al., 2017; Zaret & Paine, 1973). Food web studies combining different approaches (e.g. Valverde et al., 2020) are also needed to advance our understanding of the interactions between freshwater and marine species in the Panama Canal.

### 4.2 The Panama Canal as a possible invasion corridor for marine fishes

In total, we detected 24 marine fish species inside the Canal, some close to the Atlantic/Pacific locks near their ocean of origin, but others on the opposite ends of the Canal suggesting that they were able to cross the lowest salinity parts of Lake Gatun near the Chagres River inflow. We also detected five known interoceanic migrants inside the Canal, three of which originate from the Atlantic side and two from the Pacific. We did not detect any sequences belonging to the Indo‐Pacific lionfish (*Pterois volitans*), an invasive species in the Caribbean (Green et al., 2012). Non‐native lionfish occur near the Atlantic entrance of the Canal and there is concern that this species could invade the Eastern Pacific by crossing through the Panama Canal (Castellanos‐Galindo et al., 2020; MacIsaac et al., 2016). Interestingly, we found sequences from three Atlantic species (*Sphoeroides testudineus, Hypanus americanus* and *Hyporhamphus unifasciatus*) at the Pacific entrance of the Canal that had never been recorded before in the Eastern Pacific, albeit only in single samples. All three of these species can tolerate brackish water, but additional work is necessary to confirm their presence, especially given that they have close relatives in the Eastern Pacific. Further, ballast water released by ships after crossing the Canal is a potential source of DNA that could cause false positive identifications using the methods implemented here. Although all ships crossing the Canal are prohibited from discharging ballast water within the Canal, ballast water release at the entrances is allowed under certain circumstances (ACP, 2022).

Four species in our dataset are found in both the Pacific and the Atlantic oceans (*Diodon holocanthus, Mugil hospes, Awaous banana* and *Dajaus monticola*; Table S5). While *D. holocanthus* truly has circumtropical distribution, there is evidence suggesting that *D. monticola, A.banana* and *M. hospes* actually represent more than one species (McMahan et al., 2013, 2021; Nirchio et al., 2018). Another 17 marine detections have only genus‐level identifications, but since many genera are represented by species in both the Pacific as well as the Atlantic, their origin cannot be clearly determined. For example, the genus *Anchoa* contains multiple species distributed across the Atlantic and Pacific coasts of the Americas and sequences identified as *Anchoa* sp. could derive from *Anchoa parva* (Atlantic) or *Anchoa ischana* (Pacific). This represents a key limitation of eDNA metabarcoding as the short DNA fragments that are generated may not provide the taxonomic resolution to discriminate closely related species (Collins et al., 2019). Thus, it is possible that there are more species that have successfully entered the Canal that we cannot distinguish from congeners on the other side of the Isthmus.

Currently, there is little information about the fate of marine fishes entering the Canal, but the risk of interoceanic invasions could be increasing as more marine species are found inside of Lake Gatun and Culebra Cut. If interoceanic establishment does occur, the ecological and evolutionary consequences are diverse and potentially include hybridization events (e.g. between the non‐native Atlantic *H. americanus* and native Pacific *H. longus*) or negative impacts on native species communities due to competition, predation (e.g. feeding impact of *M. atlanticus*) and parasite transfer. More work is needed to determine if the detected marine species, of which we detected the genetic material in freshwater environments, can survive and establish within the Canal. Analysis of the isotopic composition of scales (Seeley & Walther, 2018) or otoliths (Shirai et al., 2018) is a novel technique which could be used to investigate the salinity histories of fish to determine how much of their life‐cycle is spent in the fresh waters of the Canal. Additional sampling using integrated morphological and molecular genetic approaches to identify the fish fauna will also be needed in areas outside the entrances of the Canal to confirm that interoceanic establishment has occurred. Information from sport fishing operators and artisanal fishers has previously been used to determine the distribution of the Atlantic species *M. atlanticus* in the Eastern Pacific (Castellanos‐Galindo et al., 2019; Neira et al., 2016) and would be an important data source to combine with the methods used here for future monitoring efforts.

The fact, that only four confirmed interoceanic establishments have occurred to date, highlights the effectiveness of existing dispersal barriers in the Panama Canal, such as the multiple lock system and low salinities in Lake Gatun. However, it was predicted that the recent expansion of the Canal would lead to an increase in the salinity of the Panama Canal by allowing more salt water to enter Lake Gatun and Culebra Cut (Wijsman, 2013) and surface salinity measurements support that this could be occurring (Castellanos‐Galindo et al., 2020; Jones & Dawson, 1973; Jongeling et al., 2008). Depth profiles at two sites inside of Lake Gatun and close to the Atlantic locks show salinities of up to 0.59 ppt at 20 m depth (unpublished data; G. Castellanos‐Galindo 2019/2020), suggesting that seawater entering through the locks may get concentrated in the deeper areas of Lake Gatun due to its higher density. This effect may be more pronounced near the Atlantic locks as water enters directly into the wide body of Lake Gatun in contrast to the Pacific locks which open into the narrow Culebra Cut. Marine fishes could use these higher salinity regions as a refuge from the effects of exposure to freshwater conditions. If the freshwater barrier consisting of Lake Gatun and the Culebra Cut is further compromised, biotic exchange through the Panama Canal may increase.

### 4.3 Towards efficient monitoring: Comparing traditional and eDNA‐based surveys

When comparing the results from gillnet and eDNA surveys, we observed differences in the number and type of fish species detected. Species accumulation curves showing species richness dependent on the number of sampling sites (Figure 5) did not plateau for either sampling technique, indicating that more species would likely be detected if more sites were sampled. Previous studies comparing communities described with traditional methods (e.g. trawls, visual surveys) and eDNA metabarcoding have shown that the two approaches often produce results that overlap to some extent, but not completely (Fraija‐Fernández et al., 2020; Nguyen et al., 2020; Thomsen et al., 2012) but all studies, including this one, demonstrate the power of using an integrated approach. Factors known to influence the species composition of gillnet surveys are mesh sizes and setup of the nets (i.e. proximity to shore, depth, duration of deployment), which may limit the likelihood of catching benthic and/or small fish species. Indeed, many of the 15 fish species, which were detected with eDNA metabarcoding but not with gillnets only reach a body size of 15 cm (e.g. *Astyanax panamensis*, *Atherinella chagresi*, *Gambusia holbrooki*). To overcome this methodological limitation, different net types (e.g. beach seines, trap nets), mesh sizes and setup approaches could be combined to better capture the full range of fish sizes. However, this increases the associated sampling effort accordingly (Lapointe et al., 2006).

Although we detected more fish species with eDNA metabarcoding than gillnetting at the 11 sites where both techniques were implemented, nine species caught with gillnets were not identified with eDNA. As our water samples were collected at the time of net retrieval, it is perhaps surprising to miss so many species at these sites. Failures to detect expected species in eDNA studies, or false negatives, are typically due to methodological issues (e.g. low DNA concentrations, primer mismatches, PCR inhibitors and low marker sensitivity, incomplete reference databases; (Ficetola et al., 2015)). In this study, we chose to target the COI locus since the corresponding reference database is almost complete (89% coverage) for fish species previously found in the Canal. Recent studies advocate the use alternative regions, such as the 12 S or 16 S ribosomal rRNA loci, arguing that metabarcoding using COI primers often displays low reproducibility (e.g. Collins et al., 2019; Zhang et al., 2020). However, as few of the fishes that we expected to find have been sequenced for these loci (only 55% (12 S) and 71% (16 S) sequenced; NCBI 1st March 2022), it is unlikely that the use of an alternative locus would have improved our ability to describe the fish community. Spatial heterogeneity and low concentrations of eDNA in the water may have affected our ability to detect fishes (Brys et al., 2020). Most species caught with gillnets but not identified with eDNA, are fast‐moving, pelagic species (e.g. *Caranx latus*, *Elops smithi*). In general, eDNA can be rapidly dispersed by vertical/horizontal transport (e.g. Harrison et al., 2019) and exposure to UV radiation, acidity, heat and nuclease enzymes are known to cause rapid degradation of eDNA (Dejean et al., 2011; Pilliod et al., 2014; but see Mächler et al., 2018). Suspended sediment in the water, originating from the ongoing dredging in the Canal to maintain sufficient depth of the main shipping channel, may have also influenced the quality of our DNA extractions as suspended organic material leads to filter clogging and potentially inhibits PCR through the presence of tannins and/or humic acids (Jane et al., 2015; Opel et al., 2010). Internal PCR controls can be used to test for inhibition and we recommend their integration into future metabarcoding studies (Loeza‐Quintana et al., 2020 and references therein). Finally, the selection of the primer set can lead to inconsistent amplification of DNA due to primer mismatches or untargeted amplification (Collins et al., 2019; Zhang et al., 2020). We observed multiple bands in the majority of samples amplified with our F3 primer combination, indicating that these primers were not fish specific. We recommend that future studies use several PCR technical replicates to address issues of stochasticity (Ficetola et al., 2015), especially when COI primers are used to study fish communities (Collins et al., 2019).

## 5 CONCLUSIONS

This study represents the most comprehensive attempt to characterize the fish community and detect marine fishes in the Panama Canal since its recent expansion. Both eDNA metabarcoding and traditional gillnetting revealed an increase in the number of marine species detected at several sites along the Canal, including the central portions of Lake Gatun. The observed changes in the fish community of the Panama Canal may result from salinity increases associated with the recent expansion of the Canal, but continued monitoring is needed to reliably track community shifts on the scale of the entire Canal over time. Additional studies are also needed to better understand the ecological consequences of marine fishes entering and possibly establishing populations in the Canal. As environmental conditions change in the Panama Canal, extensive and frequent eDNA sampling may provide an early warning system for invasion events by detecting species prior to successful establishment and could ultimately inform management practices.

## AUTHOR CONTRIBUTIONS

**Lennart Schreiber:** Conceptualization (equal); funding acquisition (equal); investigation (equal); writing – original draft (lead); writing – review and editing (lead). **Gustavo Adolfo Castellanos‐Galindo:** Conceptualization (lead); funding acquisition (equal); investigation (equal); project administration (equal); resources (equal); supervision (equal); writing – review and editing (equal). **D Ross Robertson:** Conceptualization (equal); investigation (equal); resources (equal); supervision (equal); writing – review and editing (equal). **Mark Torchin:** Conceptualization (equal); funding acquisition (equal); project administration (equal); supervision (equal); writing – review and editing (equal). **Karina Chavarria:** Methodology (equal); supervision (equal). **Silke Laakmann:** Conceptualization (supporting); methodology (supporting); validation (equal); visualization (equal); writing – review and editing (equal). **Kristin Saltonstall:** Conceptualization (equal); funding acquisition (equal); investigation (equal); methodology (equal); project administration (equal); resources (lead); supervision (lead); visualization (equal); writing – review and editing (equal).

## CONFLICT OF INTEREST

The authors declare that they have no conflicts of interest.

### OPEN RESEARCH BADGES

This article has earned an Open Data badge for making publicly available the digitally‐shareable data necessary to reproduce the reported results. The data is available at [http://doi.org/10.25573/data.14925360].

## Supporting information


Appendix S1:

## Data Availability

Raw sequencing data files and associated data are openly available in figshare at http://doi.org/10.25573/data.14925360.
