# Correction to “Environmental DNA (eDNA) reveals potential for interoceanic fish invasions across the Panama Canal”

**DOI:** 10.1002/ece3.10697

**Published:** 2023-11-13

Schreiber, L., Castellanos‐Galindo, G. A., Robertson, D. R., Torchin, M., Chavarria, K., Laakmann, S., and Saltonstall, K. (2023). Environmental DNA (eDNA) reveals potential for interoceanic fish invasions across the Panama Canal. *Ecol. Evol*. 13, e9675.

In this article, we reported the detection of eDNA sequences indicating the occurrence of five species of marine and fresh/brackish‐water fishes known from the Caribbean coast in water samples taken immediately outside the Pacific entrance to the Panama Canal. However, further evaluation of the CO1 sequences on BOLD kindly performed at our request by Benjamin Victor (Guy Harvey Research Institute, Nova Southeastern University; personal communication, July 9, 2023) shows that in two of those cases, the detected eDNA sequences were misidentified due to erroneous identifications in BOLD, and, in another two cases, identifications on a higher taxonomic level are more appropriate than the species‐level identifications we reported. Sequences we assigned to *Hypanus americanus* should have been identified as the Pacific species *Hypanus longus*. The erroneous entry on BOLD that we used as a reference is based on a misidentified specimen of *H. longus* from a Guatemalan fish market. In the case of *Awaous banana*, it is now recognized that there are two sister species, one in rivers on the Pacific slope (*A. transandeanus*) and one in Atlantic‐slope rivers (*A. banana*) (McMahan et al., 2021). However, because some reference entries on BOLD have not been updated in relation to this taxonomic split, we failed to assign our Pacific sequences to *A. transandeanus*. As a part of this erratum, we identified mislabeled *A. banana* BOLD references and corrected all entries for which the authors have editing rights to *A. transandeanus*. Finally, a closer inspection of the assignments of another two species (*Sphoeroides testudineus* and *Hyporhamphus unifasciatus*) shows that our eDNA sequences do not differ sufficiently from sequences of other closely related species to allow for the reliable species‐level assignment. Consequently, sequences assigned to *S. testudineus* should be identified as *Sphoeroides* sp., while those assigned to *H. unifasciatus* should be *Hyporhampus* sp.

We apologize for these errors.
