# Supplementary material for: Environmental DNA (eDNA) reveals potential for interoceanic fish invasions across the Panama Canal
Source: Ecol Evol. 2023 Jan 29;13(1):e9675. doi: 10.1002/ece3.9675 (PMC9884569; doi:10.1002/ece3.9675)
Supplement: Supplementary file 1 — Appendix S1: [file ECE3-13-e9675-s001.docx]

# **Supplemental Material**

**Environmental DNA (eDNA) reveals potential for interoceanic fish invasions across the Panama Canal**

Lennart Schreiber^1,2*^, Gustavo Castellanos-Galindo^1,3^, D. Ross Robertson^1^, Mark Torchin^1^, Karina Chavarria^1^, Silke Laakmann^4,5^, Kristin Saltonstall^1^

^1^Smithsonian Tropical Research Institute, Balboa, Panamá

^2^Faculty of Biology & Chemistry, University of Bremen, Bremen, Germany

^3^Leibniz Institute of Freshwater Ecology and Inland Fisheries (IGB), Berlin, Germany

^4^Helmholtz Institute for Functional Marine Biodiversity at the University of Oldenburg (HIFMB), Oldenburg, Germany

^5^Alfred-Wegener-Institute, Helmholtz Centre for Polar and Marine Research, Bremerhaven, Germany

*Corresponding author

Text

*Ambiguous identifications*

1. *Eleotris amblyopsis*: previously reported as *E. pisonis*, but should be referred to as *E. amblyopsis* or *E. perniger* according to Pezold and Cage (2002). Known distributions of *E. amblyopsis* and *E. perniger* indicate that individuals captured in the Panama Canal are most likely *E. amblyopsis* (Pezold and Cage, 2002).
2. *Mugil* sp.: the sequences we detected match barcodes corresponding to *Mugil cephalus*, *Mugil curema* and *Mugil setosus*. However, the presence of cryptic species in this genus is known (Durand and Borsa, 2015) and a consensus on the correct naming of all species to date does not exist (e.g. *Mugil* sp. O as part of the *Mugil curema* species complex (Durand and Borsa, 2015) is referred to as *Mugil setosus* by Britzke et al., 2019). We therefore decided to refrain from an identification to species level and recommend continued work on this genus.
3. *Micropogonias* sp.: detected sequences match both the Pacific (*M. altipinnis*) as well as the Atlantic (*M. undulatus*) species. However, when constructing a phylogenetic tree of barcodes available for both species on BOLD, we encountered a high degree of overlap or rather an entanglement of sequence records. Again, we refrained from an identification to species level and recommend continued work on this genus.

References:

Britzke, R., Menezes, N.A., and Nirchio, M. (2019). Redescription of Mugil setosus Gilbert 1892 with comments on the occurrence of Mugil curema Valenciennes 1836 in the Pacific Ocean (Teleostei: Perciformes: Mugilidae). Zootaxa *4671*, 396–406.

Durand, J.-D., and Borsa, P. (2015). Mitochondrial phylogeny of grey mullets (Acanthopterygii: Mugilidae) suggests high proportion of cryptic species. C. R. Biol. *338*, 266–277.

Pezold, F.L., and Cage, B. (2002). A review of the spinycheek sleepers, genus Eleotris (Teleostei: Eleotridae), of the Western Hemisphere, with comparison to the West African species. Tulane Stud. Zool. Bot. 19–63.

Figures

**Figure S1** Rarefaction curves of all samples as a function of sampling depth. Only ASVs assigned to fish species with >10 reads were considered.


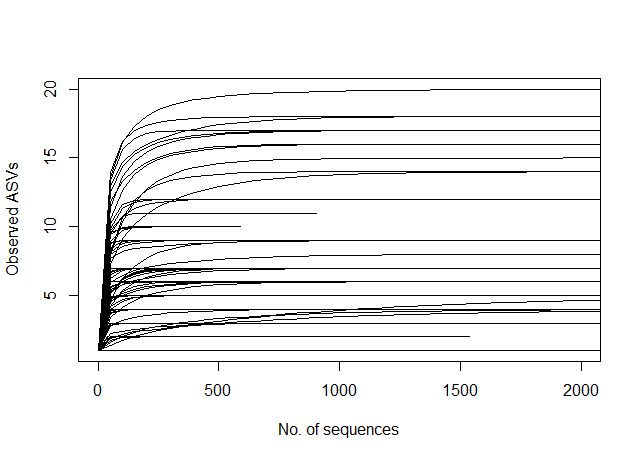


Tables

**Table S1** Full site and sampling information. Water for eDNA processing was collected at all sites. At the subset of sites where we conducted gillnet surveys, seven nets were set in the evening and retrieved early in the morning of the next day (indicated by (N)) or nets were set around midday and retrieved after 2 hours (indicated by (D)). Rio Chagres is marked with an asterisk because this site is located upstream of the inflow of Rio Chagres and is thus technically not part of the Canal. T = water temperature. The last column indicates whether sampling sites were accessed by vessel (V) or foot (F).

| Site No. & Name | Canal Section | Latitude | Longitude | Gillnet survey | eDNA | T [°C] | Salinity [ppt] | Accessed |
| --- | --- | --- | --- | --- | --- | --- | --- | --- |
| 1. Playa Farfan | Pacific | 8°56.1321 N | 79°33.6354 W |  | X | 28.2 | 26.24 | V |
| 2. Puente Americas | Pacific | 8°56.6079 N | 79°33.9712 W |  | X | 28.2 | 25.33 | V |
| 3. Balboa Port | Pacific | 8°57.4290 N | 79°34.1946 W |  | X | 28.2 | 24.52 | V |
| 4. Muelle Taller | Miraflores | 9°00.5155N | 79°35.7906W |  | X | 28.6 | 0.47 | F |
| 5. Cocodrilos | Miraflores | 9°00.9606N | 79°36.5940W |  | X | 29.0 | 1.18 | F |
| 6. Paraiso | Culebra Cut | 9°01.4793N | 79°37.2138W |  | X | 29.0 | 0.31 | F |
| 7. Culebra Cut | Culebra Cut | 9°04.5792N | 79°40.5657W |  | X | 28.5 | 0.08 | F |
| 8. Rio Chagres* | Rio Chagres* | 9°07.5322N | 79°41.5495W |  | X | 27.9 | 0.07 | F |
| 9. STRI Gamboa Dock | Culebra Cut | 9°07.1178N | 79°42.9036W |  | X | 28.8 | 0.12 | F |
| 10. BCI1 | Lake Gatun (C) | 9°08.4132 N | 79°50.0968 W |  | X | 30.2 | 0.27 | V |
| 11. Las Pavas1 | Lake Gatun (C) | 9°08.1750 N | 79°52.4472 W | X (D) | X | 29.5 | 0.26 | V |
| 12. Las Pavas2 | Lake Gatun (C) | 9°08.2413 N | 79°52.8785 W | X (D) | X | 29.6 | 0.25 | V |
| 13. BCI2 | Lake Gatun (C) | 9°10.0165N | 79°50.0562W |  | X | 29.3 | 0.26 | V |
| 14. BCI3 | Lake Gatun (C) | 9°10.1766N | 79°49.8888W |  | X | 29.4 | 0.27 | V |
| 15. BCI4 | Lake Gatun (C) | 9°10.1772N | 79°49.2807W |  | X | 29.5 | 0.23 | V |
| 16. Ahuyama | Lake Gatun (D) | 9°06.0294 N | 80°00.0567 W | X (N) | X | 28.9 | 0.19 | V |
| 17. Rancho Gatun | Lake Gatun (D) | 9°05.9964 N | 80°00.4021 W | X (N) | X | 28.8 | 0.19 | V |
| 18. Isla Caiman | Lake Gatun (D) | 9°05.4822 N | 79°59.5668 W | X (N) | X | 29.1 | 0.19 | V |
| 19. Escobal | Lake Gatun (A) | 9°11.5215 N | 79°57.0840 W | X (N) | X | 29.2 | 0.4 | V |
| 20. Punta Chivos | Lake Gatun (A) | 9°13.4136 N | 79°56.5530 W | X (N) | X | 29.5 | 0.43 | V |
| 21. Isla Bamboo | Lake Gatun (A) | 9°14.6826 N | 79°56.0764 W | X (N) | X | 29.4 | 0.48 | V |
| 22. Isla Guarapo | Lake Gatun (A) | 9°14.8908 N | 79°55.7835 W | X (N) | X | 29.6 | 0.48 | V |
| 23. Isla Limon | Lake Gatun (B) | 9°15.3018 N | 79°49.6921 W |  | X | 30.5 | 0.15 | V |
| 24. Puente Ferrocarril | Lake Gatun (B) | 9°15.3819 N | 79°51.0219 W | X (D) | X | 29.9 | 0.16 | V |
| 25. Isla Represa | Lake Gatun (B) | 9°15.7974 N | 79°51.9519 W | X (D) | X | 30.3 | 0.31 | V |
| 26. Canal Frances | Atlantic | 9°17.6106N | 79°55.2720W |  | X | 28.9 | 25.72 | F |
| 27. Puente Atlantico | Atlantic | 9°18.2145N | 79°55.2648W |  | X | 29.1 | 27.47 | F |
| 28. Limon Bay | Atlantic | 9°21.0637N | 79°56.8002W |  | X | 29.2 | 30.11 | F |

**Table S2 DNA concentrations grouped by sampling site.** Six 1 L replicates were collected at each sampling site, of which four were processed using the DNeasy PowerWater extraction kit (Qiagen) and two were processed using a modified CTAB protocol (Doyle and Doyle, 1987). The mean values of NanoDrop measurements are compiled for each sampling site and corresponding standard deviations were calculated. Some replicates were lost in the extraction process (e.g. 4. Muelle Taller (CTAB)).

| **Location** | **Extraction** | **260/280 (± sd)** | **260/230 (± sd)** | **ng/µL (± sd)** |
| --- | --- | --- | --- | --- |
| 1. Playa Farfan | PW (4) | 1.9 (± 0.05) | 1.15 (± 0.52) | 42.38 (± 5.07) |
|  | CTAB (2) | 2.01 (± 0.01) | 1.81 (± 0.06) | 114.75 (± 11.1) |
| 2. Puente Americas | PW (4) | 1.73 (± 0.29) | 0.64 (± 0.58) | 21.33 (± 21.17) |
|  | CTAB (2) | 1.99 (± 0.01) | 1.72 (± 0.04) | 101.25 (± 1.48) |
| 3. Balboa Port | PW (4) | 2 (± 0.51) | 1.21 (± 1.03) | 18.38 (± 18.94) |
|  | CTAB (2) | 1.9 (± 0.01) | 1.4 (± 0.1) | 64 (± 0) |
| 4. Muelle Taller | PW (4) | 1.83 (± 0.13) | 1.18 (± 1.02) | 28 (± 37.21) |
|  | CTAB (0) |  |  |  |
| 5. Cocodrilos | PW (4) | 1.74 (± 0.28) | 1.54 (± 0.7) | 89.53 (± 92.21) |
|  | CTAB (2) | 1.89 (± 0.01) | 0.77 (± 0.28) | 66.65 (± 18.03) |
| 6. Paraiso | PW (4) | 1.41 (± 0.58) | 1.02 (± 0.8) | 28.15 (± 30.44) |
|  | CTAB (2) | 1.89 (± 0.02) | 1.28 (± 0.34) | 99.25 (± 46.74) |
| 7. Culebra Cut | PW (4) | 1.88 (± 0.03) | 1.82 (± 0.45) | 66.18 (± 22.25) |
|  | CTAB (2) | 1.88 (± 0.02) | 1.51 (± 0) | 108.2 (± 9.9) |
| 8. Rio Chagres | PW (0) |  |  |  |
|  | CTAB (6) | 1.9 (± 0.06) | 1.24 (± 0.35) | 123.42 (± 82.12) |
| 9. STRI GD | PW (0) |  |  |  |
|  | CTAB (6) | 1.87 (± 0.03) | 1.02 (± 0.16) | 64.37 (± 10.6) |
| 10. BCI1 | PW (4) | 1.92 (± 0.1) | 1.17 (± 0.59) | 21.6 (± 3.32) |
|  | CTAB (2) | 2 (± 0.11) | 1.23 (± 0.52) | 49.25 (± 2.76) |
| 11. Las Pavas1 | PW (4) | 1.96 (± 0.02) | 1.57 (± 0.83) | 36.5 (± 4.07) |
|  | CTAB (2) | 1.93 (± 0.04) | 1.21 (± 0.17) | 86.75 (± 31.18) |
| 12. Las Pavas2 | PW (4) | 1.92 (± 0.03) | 1.87 (± 0.26) | 53.73 (± 7.94) |
|  | CTAB (2) | 1.95 (± 0.01) | 1.54 (± 0.14) | 155.45 (± 21) |
| 13. BCI2 | PW (4) | 1.57 (± 0.3) | 1.05 (± 0.66) | 10.13 (± 5.71) |
|  | CTAB (2) | 1.9 (± 0.04) | 1.06 (± 0.33) | 36.25 (± 8.7) |
| 14. BCI3 | PW (4) | 1.77 (± 0.22) | 0.44 (± 0.37) | 9.63 (± 3.51) |
|  | CTAB (2) | 1.9 (± 0.11) | 0.67 (± 0.26) | 29 (± 6.22) |
| 15. BCI4 | PW (4) | 1.69 (± 0.49) | 0.91 (± 0.78) | 10.25 (± 9.74) |
|  | CTAB (2) | 1.8 (± 0.06) | 1 (± 0.07) | 84.8 (± 75.24) |
| 16. Ahuyama | PW (4) | 1.89 (± 0.03) | 1.22 (± 0.78) | 25.45 (± 7.55) |
|  | CTAB (2) | 1.89 (± 0.03) | 0.76 (± 0.11) | 92.8 (± 10.47) |
| 17. Rancho Gatun | PW (4) | 1.97 (± 0.08) | 0.77 (± 0.48) | 23.45 (± 4.74) |
|  | CTAB (2) | 1.95 (± 0) | 0.93 (± 0) | 112.7 (± 0) |
| 18. Isla Caiman | PW (4) | 1.9 (± 0.1) | 1.45 (± 0.68) | 28.93 (± 2.44) |
|  | CTAB (2) | 1.88 (± 0.01) | 0.9 (± 0.02) | 86.4 (± 24.61) |
| 19. Escobal | PW (4) | 1.87 (± 0.05) | 0.59 (± 0.87) | 24.25 (± 3.72) |
|  | CTAB (2) | 1.93 (± 0.06) | 0.95 (± 0.23) | 55.4 (± 4.81) |
| 20. Punta Chivos | PW (4) | 1.84 (± 0.12) | -0.39 (± 1.92) | 22.1 (± 2.53) |
|  | CTAB (2) | 1.92 (± 0.01) | 0.86 (± 0.04) | 54.55 (± 2.9) |
| 21. Isla Bamboo | PW (4) | 1.85 (± 0.13) | 0.42 (± 0.37) | 23.33 (± 2.18) |
|  | CTAB (2) | 1.96 (± 0.04) | 0.93 (± 0.15) | 54.5 (± 0.85) |
| 22. Isla Guarapo | PW (4) | 1.94 (± 0.09) | -0.29 (± 1.59) | 22.2 (± 1.52) |
|  | CTAB (2) | 1.91 (± 0.1) | 0.97 (± 0.15) | 43.5 (± 21.64) |
| 23. Isla Limon | PW (4) | 1.92 (± 0.07) | 1.23 (± 0.71) | 33.45 (± 2.66) |
|  | CTAB (2) | 1.87 (± 0.04) | 0.97 (± 0.12) | 118.3 (± 13.01) |
| 24. Puente Ferrocarril | PW (4) | 1.97 (± 0.12) | 0.26 (± 2.3) | 22.4 (± 3.19) |
|  | CTAB (2) | 1.8 (± 0) | 0.79 (± 0) | 94.2 (± 0) |
| 25. Isla Represa | PW (4) | 1.86 (± 0.1) | 1.34 (± 0.73) | 20 (± 3.53) |
|  | CTAB (2) | 1.9 (± 0.04) | 0.8 (± 0.08) | 97.6 (± 8.49) |
| 26. Canal Frances | PW (4) | 1.82 (± 0.16) | 1.19 (± 0.63) | 16.45 (± 4.31) |
|  | CTAB (2) | 1.91 (± 0.01) | 1.62 (± 0.04) | 65.1 (± 2.26) |
| 27. Puente Atlantico | PW (4) | 1.85 (± 0.09) | 0.81 (± 0.82) | 28.73 (± 2.6) |
|  | CTAB (2) | 1.88 (± 0.04) | 1.44 (± 0.05) | 52 (± 0.71) |
| 28. Limon Bay | PW (4) | 1.64 (± 0.22) | 0.3 (± 0.33) | 5.93 (± 3.08) |
|  | CTAB (2) | 1.71 (± 0.21) | 0.63 (± 0.05) | - 1. ± 4.88) |

**Table S3 PCR conditions for metabarcoding fish eDNA.**

| PCR reaction components | PCR1 | PCR2 |
| --- | --- | --- |
| Reaction volume (µL) | 12.5 | 10 |
| PCR buffer (µL) | 6.25  (KAPA3G Plant PCR 2X Master Mix) | 5  ( Platinum 2X Master Mix) |
| H_2_O (µL) | 3.9 | 2 |
| Taq Polymerase (µL) | 0.1  (KAPA3G Plant DNA Polymerase) | - |
| Forward primers (µL) | 0.125  (AquaF2/AquaF3) | 1  (Illumina adapters/Index barcodes) |
| Reverse primers (µL) | 0.125  (C_FishR1) | 1  (Illumina adapters/Index barcodes) |
| DNA template (µL) | 2 | - |
| PCR1 product (µL) | - | 1 |
| **Cycle conditions** | 2 min at 96°C  40 sec at 94°C  30 sec at 51°C  30 sec at 72°C  5 min at 72°C  40 cycles | 3 min at 94°C  30 sec at 94°C  30 sec at 50°C  40 sec at 72°C  5 min at 72°C  10 cycles |
| **PCR1 Primers with 10mM concentration (reference in brackets).** Primers were modified to include partial Illumina adapters on their 5’ ends.  Forward:  AquaF2: ATCACRACCATCATYAAYATRAARCC (Ivanova et al., 2012)  AquaF3: CCAGCCATTTCNCARTACCARACRCC (Valdez-Moreno et al., 2019)  Reverse:  C_FishR1 (FR1d:FishR2; 1:1) (Ivanova et al., 2007)  FR1d: ACCTCAGGGTGTCCGAARAAYCARAA  FishR2: ACTTCAGGGTGACCGAAGAATCAGAA | | |

**Table S4 Number of reads remaining at different steps of the bioinformatics workflow.** Lab, field and extraction blanks are marked in bold (not included in the calculation of total reads).

| Sample | Total reads | filtered | denoisedF | denoisedR | merged | nonchim | only fish with >10 reads/ASV |
| --- | --- | --- | --- | --- | --- | --- | --- |
| Ahu-I | 9854 | 4364 | 4247 | 4224 | 4179 | 3806 | 163 |
| Ahu-II | 12220 | 8019 | 7905 | 7868 | 7854 | 7449 | 179 |
| Ahu-III | 14673 | 9166 | 8988 | 8914 | 8744 | 7972 | 0 |
| Ahu-IV | 10298 | 5105 | 4972 | 4903 | 4815 | 4284 | 75 |
| Ahu-V | 14103 | 8235 | 8128 | 8123 | 8076 | 7749 | 147 |
| Ahu-VI | 15839 | 9728 | 9639 | 9595 | 9557 | 9171 | 52 |
| B-P-I-1 | 10765 | 5981 | 5804 | 5720 | 5407 | 5297 | 904 |
| B-P-I-2 | 18314 | 4119 | 4075 | 4058 | 3987 | 3857 | 452 |
| B-P-II-1 | 9961 | 6500 | 6335 | 6286 | 6168 | 6072 | 3176 |
| B-P-II-2 | 31387 | 15680 | 15534 | 15536 | 15440 | 15055 | 4260 |
| B-P-III-1 | 15535 | 2197 | 2181 | 2183 | 2178 | 1735 | 0 |
| B-P-III-2 | 28385 | 97 | 40 | 19 | 19 | 8 | 0 |
| B-P-IV-1 | 14008 | 7752 | 7686 | 7695 | 7657 | 6906 | 0 |
| B-P-IV-2 | 23226 | 68 | 39 | 39 | 39 | 16 | 0 |
| B-P-V-1 | 11188 | 5330 | 5193 | 5121 | 4490 | 4342 | 1999 |
| B-P-V-2 | 19716 | 988 | 930 | 928 | 908 | 866 | 96 |
| B-P-VI-1 | 17718 | 15985 | 15864 | 15826 | 15743 | 15727 | 15058 |
| B-P-VI-2 | 27494 | 15563 | 15471 | 15418 | 15338 | 15077 | 1536 |
| BCI1I-1 | 40977 | 23003 | 22761 | 22788 | 22430 | 22341 | 195 |
| BCI1I-2 | 36114 | 6425 | 6354 | 6344 | 6035 | 5484 | 0 |
| BCI1II-1 | 25423 | 15630 | 15487 | 15483 | 15253 | 15209 | 108 |
| BCI1II-2 | 19872 | 11589 | 11491 | 11484 | 11031 | 11031 | 0 |
| BCI1III-1 | 19211 | 8996 | 8843 | 8838 | 8410 | 8344 | 135 |
| BCI1III-2 | 10336 | 978 | 948 | 938 | 307 | 304 | 0 |
| BCI1IV-1 | 21113 | 12579 | 12465 | 12440 | 12188 | 11936 | 52 |
| BCI1IV-2 | 14643 | 4951 | 4916 | 4898 | 4873 | 4838 | 0 |
| BCI1V-1 | 23755 | 15199 | 15120 | 15089 | 14940 | 14903 | 0 |
| BCI1V-2 | 14612 | 2906 | 2855 | 2847 | 2152 | 2127 | 0 |
| BCI1VI-1 | 21191 | 12887 | 12807 | 12771 | 12477 | 12452 | 0 |
| BCI1VI-2 | 16312 | 6481 | 6431 | 6410 | 4907 | 4865 | 0 |
| BCI2I | 15599 | 10033 | 9947 | 9852 | 9283 | 9127 | 0 |
| BCI2II | 18903 | 13521 | 13402 | 13281 | 12957 | 12719 | 33 |
| BCI2III | 13734 | 4617 | 4522 | 4296 | 4024 | 3801 | 0 |
| BCI2IV | 9202 | 3685 | 3620 | 3564 | 3480 | 3344 | 0 |
| BCI2V | 13014 | 8488 | 8384 | 8348 | 7988 | 7525 | 215 |
| BCI2VI | 16321 | 9801 | 9708 | 9611 | 9225 | 8958 | 0 |
| BCI3I | 18135 | 13153 | 13044 | 12965 | 12539 | 12423 | 0 |
| BCI3II | 12638 | 6730 | 6647 | 6604 | 6247 | 6204 | 0 |
| BCI3III | 13823 | 6699 | 6596 | 6542 | 6117 | 6016 | 0 |
| BCI3IV | 11922 | 6012 | 5923 | 5866 | 5328 | 5288 | 0 |
| BCI3V | 15116 | 8160 | 8076 | 8041 | 7376 | 7347 | 0 |
| BCI4I | 11736 | 8251 | 8164 | 8115 | 7785 | 7735 | 0 |
| BCI4II | 11022 | 7508 | 7422 | 7407 | 7060 | 6938 | 214 |
| BCI4III | 11812 | 616 | 591 | 571 | 550 | 537 | 0 |
| BCI4IV | 9383 | 1033 | 1020 | 938 | 935 | 933 | 0 |
| BCI4V | 12281 | 7538 | 7471 | 7451 | 6908 | 6884 | 0 |
| BCI4VI | 4 | 4 | 3 | 3 | 0 | 0 | 0 |
| C-C-I-1 | 2179 | 826 | 780 | 780 | 763 | 705 | 38 |
| C-C-I-2 | 17090 | 2655 | 2620 | 2300 | 2170 | 2170 | 39 |
| C-C-II-1 | 9860 | 4025 | 3933 | 3909 | 3859 | 3531 | 24 |
| C-C-II-2 | 15960 | 953 | 902 | 649 | 556 | 540 | 0 |
| C-C-III-1 | 8523 | 4121 | 4045 | 4009 | 3971 | 3639 | 0 |
| C-C-III-2 | 23922 | 7796 | 7668 | 6295 | 6221 | 6092 | 310 |
| C-C-IV-1 | 9040 | 4460 | 4375 | 4335 | 4310 | 4054 | 28 |
| C-C-IV-2 | 15813 | 2741 | 2659 | 1981 | 1886 | 1886 | 0 |
| C-C-V-1 | 9766 | 2782 | 2731 | 2704 | 2629 | 2473 | 47 |
| C-C-V-2 | 17982 | 2008 | 1971 | 1937 | 1411 | 1395 | 0 |
| C-C-VI-1 | 10924 | 3740 | 3677 | 3628 | 3530 | 3316 | 77 |
| C-C-VI-2 | 18990 | 2180 | 2139 | 1959 | 1952 | 1943 | 77 |
| C-F-I-1 | 26113 | 9791 | 9641 | 9569 | 8673 | 8606 | 374 |
| C-F-I-2 | 12265 | 2655 | 2588 | 2591 | 2507 | 2472 | 43 |
| C-F-II-1 | 34440 | 12051 | 11918 | 11827 | 11121 | 11054 | 1072 |
| C-F-II-2 | 26580 | 1532 | 1478 | 1476 | 1472 | 1445 | 11 |
| C-F-III-1 | 23681 | 7465 | 7378 | 7311 | 6870 | 6786 | 76 |
| C-F-III-2 | 67 | 5 | 1 | 1 | 0 | 0 | 0 |
| C-F-IV-1 | 24068 | 8743 | 8595 | 8516 | 8216 | 8198 | 368 |
| C-F-IV-2 | 14590 | 2809 | 2751 | 2742 | 2718 | 2702 | 48 |
| C-F-V-1 | 25767 | 8845 | 8683 | 8601 | 7732 | 7675 | 480 |
| C-F-V-2 | 21003 | 1555 | 1504 | 1482 | 1465 | 1449 | 81 |
| C-F-VI-1 | 2 | 1 | 1 | 1 | 1 | 0 | 0 |
| C-F-VI-2 | 19242 | 2968 | 2929 | 2890 | 2757 | 2704 | 326 |
| Coc-I-1 | 31481 | 15518 | 15426 | 15400 | 15100 | 14864 | 201 |
| Coc-I-2 | 22492 | 9773 | 9669 | 9624 | 9490 | 9155 | 5952 |
| Coc-II-1 | 26653 | 23034 | 22899 | 22844 | 22370 | 21693 | 826 |
| Coc-II-2 | 21705 | 10072 | 10003 | 9840 | 9742 | 9522 | 6406 |
| Coc-III-1 | 20224 | 14092 | 13962 | 13946 | 13488 | 13183 | 474 |
| Coc-III-2 | 16355 | 2748 | 2684 | 2681 | 2326 | 2037 | 430 |
| Coc-IV-1 | 15436 | 6562 | 6512 | 6404 | 6216 | 6001 | 92 |
| Coc-IV-2 | 15837 | 2328 | 2307 | 2287 | 2270 | 2045 | 451 |
| Coc-V-1 | 20380 | 13859 | 13743 | 13711 | 12268 | 12046 | 979 |
| Coc-V-2 | 18954 | 2206 | 2179 | 2174 | 2170 | 2170 | 1156 |
| Coc-VI-1 | 2 | 2 | 1 | 1 | 0 | 0 | 0 |
| Coc-VI-2 | 16453 | 850 | 807 | 802 | 791 | 784 | 104 |
| Esc-I | 34201 | 9162 | 9003 | 9040 | 8782 | 8325 | 0 |
| Esc-II | 22705 | 10869 | 10626 | 10640 | 10311 | 9216 | 0 |
| Esc-III | 25302 | 13760 | 13667 | 13635 | 13469 | 12563 | 0 |
| Esc-IV | 19324 | 9760 | 9675 | 9633 | 9378 | 8771 | 0 |
| Esc-V | 19052 | 10727 | 10649 | 10588 | 10384 | 9702 | 81 |
| Esc-VI | 16099 | 9419 | 9322 | 9274 | 9172 | 8855 | 0 |
| I-B-I | 12901 | 6880 | 6778 | 6755 | 6419 | 6215 | 0 |
| I-B-II | 9622 | 4376 | 4308 | 4295 | 4002 | 3930 | 0 |
| I-B-III | 12115 | 6590 | 6528 | 6512 | 6273 | 6062 | 0 |
| I-B-IV | 11003 | 5486 | 5407 | 5399 | 5220 | 5015 | 68 |
| I-B-V | 11542 | 6553 | 6443 | 6423 | 6157 | 6049 | 0 |
| I-B-VI | 9970 | 5114 | 5060 | 4984 | 4686 | 4607 | 39 |
| I-C-I | 10864 | 6151 | 5969 | 5936 | 5893 | 5029 | 1442 |
| I-C-II | 12907 | 8866 | 8686 | 8684 | 8515 | 7398 | 2159 |
| I-C-III | 24073 | 20633 | 20473 | 20389 | 20265 | 19402 | 16056 |
| I-C-IV | 12558 | 7273 | 7099 | 7075 | 6947 | 5745 | 687 |
| I-C-V | 16043 | 10945 | 10831 | 10764 | 10597 | 9831 | 3013 |
| I-C-VI | 14843 | 8970 | 8774 | 8782 | 8662 | 7664 | 3341 |
| I-G-I | 12644 | 7218 | 7108 | 7133 | 6556 | 6377 | 15 |
| I-G-II | 11294 | 5873 | 5779 | 5777 | 5625 | 5480 | 0 |
| I-G-III | 8448 | 3337 | 3249 | 3273 | 3192 | 3129 | 0 |
| I-G-IV | 9188 | 3943 | 3893 | 3891 | 3842 | 3727 | 0 |
| I-G-V | 9832 | 4364 | 4308 | 4292 | 3825 | 3819 | 0 |
| I-G-VI | 9306 | 4307 | 4258 | 4243 | 3826 | 3826 | 0 |
| I-L-I | 12136 | 7797 | 7626 | 7559 | 7424 | 7375 | 205 |
| I-L-II | 10818 | 6686 | 6540 | 6515 | 6402 | 6374 | 50 |
| I-L-III | 10458 | 4917 | 4777 | 4759 | 4670 | 4599 | 80 |
| I-L-IV | 10162 | 6178 | 6053 | 6029 | 5968 | 5906 | 81 |
| I-L-V | 12710 | 9211 | 9150 | 9133 | 9077 | 8995 | 250 |
| I-L-VI | 11785 | 7673 | 7622 | 7593 | 7532 | 7490 | 462 |
| I-R-I | 16503 | 8202 | 8062 | 8009 | 7924 | 7790 | 0 |
| I-R-II | 14776 | 7041 | 6887 | 6858 | 6579 | 6434 | 0 |
| I-R-III | 15619 | 6324 | 6186 | 6160 | 6092 | 5969 | 0 |
| I-R-IV | 14121 | 5427 | 5319 | 5276 | 5174 | 5012 | 0 |
| I-R-V | 15500 | 8652 | 8571 | 8512 | 8327 | 8239 | 0 |
| I-R-VI | 16134 | 8774 | 8675 | 8646 | 8212 | 8083 | 37 |
| L-B-I-1 | 2214 | 851 | 756 | 751 | 747 | 726 | 327 |
| L-B-I-2 | 19465 | 1509 | 1461 | 1459 | 1336 | 1280 | 268 |
| L-B-II-1 | 18697 | 6832 | 6708 | 6643 | 6574 | 6283 | 2611 |
| L-B-II-2 | 19918 | 2162 | 2104 | 2095 | 1886 | 1654 | 661 |
| L-B-III-1 | 14588 | 2907 | 2813 | 2699 | 2683 | 2549 | 514 |
| L-B-III-2 | 36197 | 2928 | 2906 | 2901 | 2870 | 2865 | 0 |
| L-B-IV-1 | 18177 | 4301 | 4207 | 4167 | 4034 | 3877 | 1078 |
| L-B-IV-2 | 20806 | 4296 | 4262 | 4254 | 4160 | 4148 | 0 |
| L-B-V-1 | 22862 | 4714 | 4564 | 4516 | 4426 | 4297 | 837 |
| L-B-V-2 | 22361 | 690 | 647 | 616 | 536 | 495 | 91 |
| L-B-VI-1 | 8872 | 212 | 164 | 159 | 150 | 150 | 13 |
| L-B-VI-2 | 7862 | 80 | 51 | 44 | 17 | 15 | 0 |
| L-P-1I-1 | 15941 | 10602 | 10448 | 10409 | 10236 | 9439 | 392 |
| L-P-1I-2 | 18492 | 1577 | 1537 | 1522 | 980 | 965 | 26 |
| L-P-1II-1 | 11472 | 6744 | 6570 | 6555 | 6478 | 5995 | 106 |
| L-P-1II-2 | 20639 | 9334 | 9265 | 9262 | 8916 | 8912 | 756 |
| L-P-1III-1 | 16595 | 9102 | 8965 | 8911 | 8805 | 8122 | 279 |
| L-P-1III-2 | 171 | 15 | 1 | 1 | 0 | 0 | 0 |
| L-P-1IV-1 | 17642 | 9394 | 9233 | 9146 | 8890 | 8243 | 15 |
| L-P-1IV-2 | 26523 | 1217 | 1175 | 1166 | 796 | 773 | 0 |
| L-P-1V-1 | 23576 | 14523 | 14399 | 14327 | 13847 | 13363 | 408 |
| L-P-1V-2 | 114 | 43 | 18 | 8 | 8 | 3 | 0 |
| L-P-1VI-1 | 32123 | 7045 | 6987 | 6972 | 6806 | 6797 | 0 |
| L-P-1VI-2 | 46350 | 6018 | 5968 | 5951 | 5011 | 5011 | 192 |
| L-P-2I-1 | 7117 | 4236 | 4088 | 4086 | 3933 | 3766 | 222 |
| L-P-2I-2 | 60 | 8 | 1 | 1 | 0 | 0 | 0 |
| L-P-2II-1 | 8298 | 4896 | 4787 | 4748 | 4634 | 4492 | 857 |
| L-P-2II-2 | 22220 | 3597 | 3532 | 3285 | 3194 | 3183 | 1007 |
| L-P-2III-1 | 9898 | 4493 | 4408 | 4366 | 4249 | 4101 | 466 |
| L-P-2III-2 | 25166 | 4856 | 4797 | 4786 | 4101 | 3944 | 146 |
| L-P-2IV-1 | 8529 | 4968 | 4849 | 4845 | 4777 | 4660 | 945 |
| L-P-2IV-2 | 25322 | 6595 | 6558 | 6523 | 5761 | 5548 | 178 |
| L-P-2V-1 | 10819 | 6326 | 6200 | 6184 | 6025 | 5912 | 1202 |
| L-P-2V-2 | 132 | 10 | 1 | 2 | 0 | 0 | 0 |
| L-P-2VI-1 | 11475 | 6217 | 6104 | 6110 | 6041 | 5939 | 1730 |
| L-P-2VI-2 | 25591 | 1235 | 1194 | 1165 | 1156 | 1152 | 519 |
| M-T-I-1 | 15482 | 11007 | 10888 | 10870 | 10385 | 10186 | 2846 |
| M-T-I-2 | 22744 | 7801 | 7745 | 7703 | 7489 | 7288 | 2294 |
| M-T-II-1 | 10080 | 6930 | 6815 | 6766 | 6425 | 6314 | 1660 |
| M-T-II-2 | 18528 | 566 | 527 | 452 | 448 | 446 | 58 |
| M-T-III-1 | 10732 | 36 | 26 | 26 | 26 | 26 | 0 |
| M-T-III-2 | 21381 | 27 | 4 | 3 | 3 | 3 | 0 |
| M-T-IV-1 | 5879 | 551 | 541 | 537 | 522 | 522 | 0 |
| M-T-IV-2 | 17926 | 28 | 3 | 2 | 0 | 0 | 0 |
| P-Am-I-1 | 25099 | 15301 | 15098 | 14955 | 14665 | 14428 | 5669 |
| P-Am-I-2 | 19576 | 1353 | 1307 | 1306 | 1306 | 1296 | 229 |
| P-Am-II-1 | 27547 | 17373 | 17156 | 17067 | 16768 | 16477 | 3520 |
| P-Am-II-2 | 35902 | 12306 | 12243 | 12231 | 12208 | 11854 | 2405 |
| P-Am-III-1 | 24186 | 7991 | 7891 | 7864 | 6718 | 6621 | 0 |
| P-Am-III-2 | 21038 | 285 | 233 | 218 | 218 | 188 | 0 |
| P-Am-IV-1 | 36036 | 23750 | 23686 | 23626 | 23590 | 23389 | 9866 |
| P-Am-IV-2 | 16425 | 6040 | 6025 | 6016 | 5995 | 5796 | 0 |
| P-Am-V-1 | 28072 | 14244 | 14063 | 13911 | 12815 | 12725 | 1759 |
| P-Am-V-2 | 16493 | 4736 | 4713 | 4697 | 4661 | 4156 | 216 |
| P-Am-VI-1 | 33928 | 18297 | 18064 | 17755 | 16745 | 16501 | 3467 |
| P-Am-VI-2 | 21499 | 2786 | 2696 | 2678 | 2600 | 2565 | 164 |
| P-AtlI | 32085 | 11308 | 11119 | 11030 | 10581 | 10541 | 1399 |
| P-AtlII | 21523 | 8747 | 8519 | 8442 | 8282 | 8264 | 647 |
| P-AtlIII | 22612 | 8827 | 8620 | 8591 | 8385 | 8313 | 509 |
| P-AtlIV | 19929 | 7219 | 7029 | 6877 | 6676 | 6627 | 512 |
| P-AtlV | 18720 | 5165 | 5004 | 5000 | 4587 | 4532 | 591 |
| P-C-I | 19364 | 10548 | 10436 | 10377 | 10184 | 9728 | 0 |
| P-C-II | 19015 | 10779 | 10674 | 10662 | 10398 | 9799 | 31 |
| P-C-III | 16399 | 8711 | 8553 | 8559 | 8231 | 7742 | 0 |
| P-C-IV | 15891 | 8305 | 8196 | 8140 | 7930 | 7570 | 0 |
| P-C-V | 16222 | 8438 | 8371 | 8345 | 7776 | 7331 | 0 |
| P-C-VI | 16395 | 9318 | 9224 | 9200 | 8969 | 8636 | 0 |
| P-F-I | 33035 | 21622 | 21407 | 21380 | 21116 | 20983 | 273 |
| P-F-II | 54500 | 26608 | 26503 | 26481 | 26312 | 25698 | 182 |
| P-F-III | 34008 | 22923 | 22761 | 22711 | 22544 | 22508 | 22 |
| P-F-IV | 32237 | 21319 | 21174 | 21125 | 20963 | 20603 | 112 |
| P-F-VI | 37185 | 28850 | 28669 | 28605 | 28105 | 27885 | 220 |
| Par-I-1 | 8822 | 3722 | 3656 | 3649 | 3531 | 3421 | 2723 |
| Par-I-2 | 27477 | 8857 | 8799 | 8694 | 8676 | 8657 | 7809 |
| Par-II-1 | 6451 | 1113 | 1047 | 1020 | 914 | 844 | 198 |
| Par-II-2 | 29169 | 7749 | 7662 | 7261 | 7022 | 6527 | 640 |
| Par-III-1 | 7030 | 588 | 573 | 573 | 573 | 573 | 0 |
| Par-III-2 | 25520 | 29 | 5 | 1 | 0 | 0 | 0 |
| Par-IV-1 | 5923 | 55 | 35 | 35 | 35 | 35 | 0 |
| Par-IV-2 | 31019 | 49 | 4 | 4 | 4 | 0 | 0 |
| Par-V-1 | 8021 | 1104 | 1067 | 1065 | 911 | 900 | 340 |
| Par-V-2 | 37931 | 4851 | 4785 | 4499 | 4462 | 4194 | 2456 |
| Par-VI-2 | 41376 | 5804 | 5717 | 5648 | 5328 | 5059 | 528 |
| Pl-Fa-I-1 | 21629 | 13393 | 13156 | 12977 | 12727 | 12627 | 3184 |
| Pl-Fa-I-2 | 23328 | 4695 | 4650 | 4645 | 4621 | 4332 | 1288 |
| Pl-Fa-II-1 | 21173 | 13828 | 13576 | 13469 | 13281 | 12930 | 3917 |
| Pl-Fa-II-2 | 21949 | 5675 | 5627 | 5588 | 5562 | 5125 | 1324 |
| Pl-Fa-III-1 | 24280 | 13139 | 12918 | 12804 | 12504 | 12264 | 3355 |
| Pl-Fa-III-2 | 29058 | 2023 | 1982 | 1970 | 1959 | 1811 | 123 |
| Pl-Fa-IV-1 | 25124 | 14210 | 13998 | 13786 | 13364 | 13104 | 2929 |
| Pl-Fa-IV-2 | 40973 | 24300 | 24183 | 24107 | 23643 | 22280 | 7012 |
| Pl-Fa-V-1 | 24869 | 10672 | 10474 | 10362 | 9689 | 9595 | 1287 |
| Pl-Fa-V-2 | 23699 | 4407 | 4361 | 4346 | 4329 | 4031 | 369 |
| Pl-Fa-VI-1 | 27754 | 12466 | 12286 | 12170 | 11612 | 11558 | 2165 |
| Pl-Fa-VI-2 | 23337 | 1158 | 1104 | 1086 | 1081 | 1072 | 104 |
| R-C-I-1 | 2199 | 1484 | 1463 | 1457 | 1326 | 1321 | 48 |
| R-C-I-2 | 20908 | 8912 | 8861 | 8849 | 8675 | 8513 | 28 |
| R-C-II-1 | 8241 | 3153 | 3122 | 3111 | 2964 | 2899 | 32 |
| R-C-II-2 | 19955 | 4362 | 4299 | 4288 | 4145 | 4145 | 0 |
| R-C-III-1 | 12486 | 6726 | 6694 | 6655 | 6452 | 6383 | 145 |
| R-C-III-2 | 18725 | 2332 | 2311 | 2179 | 2168 | 2139 | 0 |
| R-C-IV-1 | 9879 | 5616 | 5554 | 5573 | 5301 | 5196 | 191 |
| R-C-IV-2 | 21062 | 10240 | 10212 | 10074 | 10050 | 10047 | 3628 |
| R-C-V-1 | 18945 | 14042 | 13915 | 13887 | 13456 | 12608 | 198 |
| R-C-V-2 | 25365 | 10550 | 10471 | 10421 | 8533 | 8389 | 120 |
| R-C-VI-1 | 17370 | 11726 | 11531 | 11513 | 11172 | 10483 | 12 |
| R-C-VI-2 | 12993 | 3651 | 3595 | 3573 | 3501 | 3483 | 0 |
| R-G-I | 5638 | 2667 | 2594 | 2584 | 2503 | 2473 | 0 |
| R-G-II | 7160 | 4198 | 4110 | 4085 | 4066 | 3998 | 112 |
| R-G-III | 8707 | 4595 | 4495 | 4474 | 4462 | 4408 | 398 |
| R-G-IV | 7985 | 4133 | 3997 | 3973 | 3943 | 3844 | 79 |
| R-G-V | 8510 | 5243 | 5087 | 5145 | 5055 | 5049 | 0 |
| STRIGDI-1 | 4749 | 1395 | 1345 | 1334 | 1230 | 1141 | 12 |
| STRIGDI-2 | 36235 | 2713 | 2665 | 2655 | 2070 | 2046 | 27 |
| STRIGDII-1 | 17493 | 5199 | 5111 | 5031 | 4686 | 4347 | 0 |
| STRIGDII-2 | 27700 | 5778 | 5686 | 5681 | 4461 | 4449 | 0 |
| STRIGDIII-1 | 14598 | 3211 | 3133 | 3133 | 2883 | 2714 | 0 |
| STRIGDIII-2 | 31947 | 2581 | 2538 | 1657 | 1648 | 1605 | 0 |
| STRIGDIV-1 | 16756 | 4789 | 4695 | 4697 | 4276 | 4014 | 50 |
| STRIGDIV-2 | 27045 | 1380 | 1316 | 884 | 866 | 845 | 0 |
| STRIGDV-1 | 17839 | 4450 | 4372 | 4337 | 4004 | 3781 | 16 |
| STRIGDV-2 | 20912 | 1814 | 1756 | 1634 | 1614 | 1578 | 0 |
| STRIGDVI-1 | 18412 | 6191 | 6072 | 6046 | 5510 | 5317 | 0 |
| STRIGDVI-2 | 23376 | 3160 | 3109 | 3069 | 2081 | 2074 | 286 |
| **LBI-1** | **8833** | **1** | **1** | **1** | **0** | **0** | **0** |
| **LBI-2** | **511** | **2** | **1** | **1** | **0** | **0** | **0** |
| **LBIII-1** | **6463** | **2** | **1** | **1** | **0** | **0** | **0** |
| **LBIV** | **2524** | **2** | **1** | **1** | **1** | **0** | **0** |
| **LBV** | **676** | **1** | **1** | **1** | **0** | **0** | **0** |
| **LBVI** | **1113** | **1** | **1** | **1** | **0** | **0** | **0** |
| **LBVIII** | **1007** | **1** | **1** | **1** | **1** | **0** | **0** |
| **FB03-12** | **3356** | **144** | **134** | **134** | **134** | **134** | **0** |
| **FB05-12** | **15162** | **312** | **292** | **292** | **292** | **292** | **0** |
| **FB11-02** | **11657** | **448** | **434** | **434** | **434** | **434** | **0** |
| **FB13-12** | **12343** | **100** | **85** | **85** | **85** | **85** | **0** |
| **FB17-01** | **18641** | **19** | **7** | **2** | **0** | **0** | **0** |
| **FB27-01** | **20143** | **108** | **83** | **83** | **83** | **83** | **0** |
| **EB10-02-1_S254** | **18120** | **3259** | **3241** | **3237** | **3235** | **3235** | **0** |
| **EB10-02-2_S264** | **9886** | **1791** | **1788** | **1787** | **1786** | **1786** | **1786** |
| **EB11-02-1_S255** | **16184** | **30** | **3** | **1** | **0** | **0** | **0** |
| **EB11-02-2_S268** | **1523** | **1** | **1** | **1** | **0** | **0** | **0** |
| **EB12-02-1_S256** | **20790** | **211** | **185** | **185** | **184** | **184** | **0** |
| **EB12-02-2_S272** | **18004** | **16832** | **16825** | **16811** | **16797** | **16797** | **0** |
| **EB13-02B_S280** | **11457** | **3** | **1** | **1** | **1** | **0** | **0** |
| **EB21-01_S271** | **2316** | **4** | **1** | **1** | **0** | **0** | **0** |
| **EB22-01_S275** | **12429** | **4** | **1** | **1** | **0** | **0** | **0** |
| **EB24-01_S279** | **1197** | **4** | **2** | **2** | **0** | **0** | **0** |
| **EB28-01-1_S251** | **3639** | **1** | **1** | **1** | **0** | **0** | **0** |
| **EB28-01-2_S283** | **1875** | **2** | **1** | **1** | **0** | **0** | **0** |
| **EB30-01-1_S252** | **14674** | **5** | **2** | **1** | **0** | **0** | **0** |
| **EB30-01-2_S286** | **262** | **4** | **4** | **4** | **0** | **0** | **0** |
| **EB31-01-1_S253** | **20636** | **17** | **2** | **1** | **0** | **0** | **0** |
| **EB31-01-2_S260** | **413** | **4** | **1** | **1** | **0** | **0** | **0** |
| Total | 4,366,344 | 1,662,365 | 1,640,054 | 1,626,515 | 1,569,422 | 1,522,768 | 179,335 |

**Table S5 Complete species list of eDNA detections.** Predicted habitats (f = freshwater, b = brackish, m = marine) and geographic ranges (A = Atlantic, P = Pacific; As = Atlantic slope, Ps = Pacific slope) were retrieved from biogeodb.stri.si.edu/caribbean and biogeodb.stri.si.edu/sftep and iucnredlist.org (individual species assessment retrieved 10/06/2020). Section code: A: Atlantic, G: Lake Gatun, C: Culebra Cut, M: Miraflores, P: Pacific. F2/F3 indicate the primer set which produced the respective sequences. Introduced species are marked in bold and Indo-Pacific species are marked with (!).

| **Species list** | | **Habitat** | **Detected at section** | **Primer** | **Total reads** |
| --- | --- | --- | --- | --- | --- |
| Ariidae | *Ariopsis seemanni* | f/b/m (P) | P | F3 | 115 |
|  | *Bagre pinnimaculatus* | f/b/m (P) | P | F3 | 138 |
| Atherinopsidae | *Atherinella chagresi* | f/b (As) | G,C,M,RC | F2,F3 | 28831 |
| Belonidae | *Strongylura exilis* | f/b/m (P) | P | F3 | 8316 |
|  | *Tylosurus acus* | m (P) | P | F3 | 10 |
| Blenniidae | *Hypleurochilus* sp*.* | f/b/m (A) | A | F3 | 144 |
|  | *Omobranchus punctatus* (!) | b | A | F3 | 236 |
|  | *Ophioblennius macclurei* | m (A) | A | F3 | 17 |
| Bythitidae | *Ogilbia* sp*.* | f/b/m (A/P) | A | F3 | 198 |
| Bryconidae | *Brycon chagrensis* | f (As) | C,M,P,RC | F2,F3 | 4382 |
| Centropomidae | *Centropomus undecimalis* | f/b/m (A) | A,G | F2 | 119 |
|  | *Centropomus unionensis* | f/b/m (P) | C | F2 | 19 |
| Chaenopsidae | *Ekemblemaria nigra* | m (A) | A | F3 | 17 |
| Chanidae | *Chanos chanos* | b/m (P) | P | F3 | 70 |
| Characidae | *Astyanax bimaculatus* | f (N/A) | C,M,RC | F3 | 148 |
|  | *Astyanax panamensis* | f (As/Ps) | G,RC | F3 | 181 |
|  | *Odontostilbe mitoptera* | f (As) | RC | F3 | 72 |
|  | *Roeboides* sp. | f (As/Ps) | RC,M | F3 | 116 |
| Cichlidae | ***Astronotus ocellatus*** | f | G,RC | F3 | 1410 |
|  | ***Cichla ocellaris*** | f | G | F3 | 112 |
|  | ***Coptodon rendalli*** | f/b | G | F3 | 124 |
|  | *Isthmoheros tuyrensis* | f (As/Ps) | RC | F3 | 45 |
|  | ***Oreochromis* sp.** | f/b | G,C,RC,M,P | F2,F3 | 16129 |
|  | ***Parachromis managuensis*** | f | G,RC | F3 | 359 |
|  | *Vieja maculicauda* | f/b (As) | G,C,M,RC | F2,F3 | 270 |
| Clupeidae | *Harengula* sp. | b/m (A/P) | A | F3 | 125 |
|  | *Jenkinsia lamprotaenia* | m (A) | A | F3 | 54 |
|  | *Opisthonema libertate* | b/m (P) | P | F2,F3 | 1027 |
|  | *Opisthonema oglinum* | b/m (A) | A | F2,F3 | 681 |
|  | *Opisthonema* sp. | b/m (A/P) | P | F3 | 631 |
| Dasyatidae | *Hypanus americanus* | f/b/m (A) | P | F3 | 345 |
| Diodontidae | *Diodon holocanthus* | b/m (A/P) | P | F3 | 74 |
| Eleotridae | *Butis koilomatodon* (!) | f/b/m | A | F3 | 182 |
|  | *Dormitator latifrons* | f/b/m (P) | G,C,M,P | F3 | 6098 |
|  | *Dormitator maculatus* | f/b/m (A) | G | F3 | 68 |
|  | *Eleotris amblyopsis* | f/b/m (A) | A | F2,F3 | 21 |
|  | *Eleotris picta* | f/b/m (P) | C,M,P | F2,F3 | 10732 |
|  | *Erotelis smaragdus* | b/m (A) | A | F3 | 27 |
|  | *Gobiomorus dormitor* | f/b/m (A) | G,C,M,P | F3 | 2897 |
|  | *Gobiomorus maculatus* | f/b/m (P) | C,M,P | F2,F3 | 429 |
|  | *Leptophilypnus fluviatilis* | f/b (As) | G | F2,F3 | 393 |
|  | *Leptophilypnus panamensis* | f | M | F2 | 20 |
| Engraulidae | *Anchoa* sp. | f/b/m (A/P) | A,G,C,M,P | F2,F3 | 38843 |
|  | *Anchovia macrolepidota* | b/m (P) | P | F3 | 68 |
|  | *Cetengraulis mysticetus* | b/m (P) | P | F2,F3 | 17815 |
| Ephippidae | *Chaetodipterus* sp. | b/m (A/P) | P | F3 | 267 |
| Erythrinidae | *Hoplias microlepis* | f (As/Ps) | C,M,RC | F3 | 66 |
| Gerreidae | *Diapterus auratus* | f/b/m (A) | A | F2 | 38 |
|  | *Diapterus brevirostris* | f/b/m (P) | C,M,P | F2 | 9361 |
|  | *Diapterus rhombeus* | f/b/m (A) | A | F2 | 369 |
|  | *Eucinostomus currani* | f/b/m (P) | C | F2,F3 | 86 |
|  | *Eucinostomus harengulus* | b/m (A) | A | F2,F3 | 1086 |
|  | *Eucinostomus jonesii* | m (A) | A | F2,F3 | 847 |
|  | *Eucinostomus melanopterus* | f/b/m (A) | A | F3 | 111 |
|  | *Eucinostomus* sp. | f/b/m (A/P) | P | F3 | 613 |
|  | *Eugerres brasilianus* | f/b/m (A) | G,C,RC | F2 | 1481 |
|  | *Eugerres plumieri* | f/b/m (A) | A | F3 | 333 |
| Gobiidae | *Awaous banana* | f/b (A) | C,M,P | F3 | 523 |
|  | *Bathygobius andrei* | f/b/m (P) | P | F3 | 43 |
|  | *Bathygobius soporator* | f/b/m (A) | A | F3 | 23 |
|  | *Coryphopterus glaucofraenum* | m (A) | A | F3 | 110 |
|  | *Ctenogobius boleosoma* | f/b/m (A) | A | F3 | 113 |
|  | *Ctenogobius manglicola* | b (P) | P | F2,F3 | 15 |
|  | *Ctenogobius sagittula* | f/b (P) | M,P | F2,F3 | 193 |
|  | *Gobionellus microdon* | f/b (P) | M | F3 | 49 |
|  | *Gobionellus oceanicus* | f/b/m (A) | G | F3 | 151 |
|  | *Gobiosoma hildebrandi* | f/b/m (A) | G,M | F2,F3 | 76 |
|  | *Gobiosoma homochroma* | f/b (P) | C | F3 | 63 |
|  | *Lophogobius cyprinoides* | f/b (A) | A | F3 | 47 |
|  | *Microgobius meeki* | b/m (A) | A | F3 | 81 |
|  | *Microgobius tabogensis* | b/m (P) | C,P | F3 | 792 |
| Haemulidae | *Anisotremus caesius* | m (P) | P | F2 | 49 |
|  | *Anisotremus surinamensis* | b/m (A) | A | F3 | 17 |
|  | *Genyatremus dovii* | b/m (P) | P | F3 | 15 |
|  | *Genyatremus pacifici* | f/b/m (P) | P | F2,F3 | 3407 |
|  | *Haemulon bonariense* | m (A) | A | F3 | 93 |
|  | *Haemulon flavolineatum* | m (A) | A | F2 | 46 |
|  | *Haemulon* sp. | b/m (A/P) | A | F2 | 661 |
|  | *Haemulon atlanticus* | b/m (A) | A | F3 | 167 |
|  | *Haemulopsis leuciscus* | f/b/m (P) | P | F2,F3 | 980 |
|  | *Orthopristis chalceus* | b/m (P) | P | F3 | 137 |
|  | *Rhencus panamensis* | b/m (P) | P | F3 | 860 |
|  | *Rhonciscus crocro* | f/b/m (A) | A | F3 | 517 |
| Hemiramphidae | *Hyporhamphus unifasciatus* | b/m (A) | P | F3 | 179 |
| Heptapteridae | *Rhamdia quelen* | f (As/Ps) | G,P | F3 | 224 |
| Kyphosidae | *Kyphosus elegans* | f/b/m (P) | P | F3 | 435 |
| Labridae | *Halichoeres poeyi* | m (A) | A | F3 | 102 |
| Labrisomidae | *Brockius nigricinctus* | m (A) | A | F3 | 64 |
|  | *Labrisomus cricota* | m (A) | A | F3 | 180 |
|  | *Labrisomus nuchipinnis* | m (A) | A | F2 | 268 |
|  | *Malacoctenus delalandii* | b/m (A) | A | F3 | 2450 |
|  | *Malacoctenus* sp. | b/m (A/P) | A | F3 | 628 |
|  | *Paraclinus fasciatus* | m (A) | A | F2,F3 | 340 |
| Loricariidae | Genus 1 sp. | f (As/Ps) | RC | F3 | 17 |
| Lutjanidae | *Lutjanus colorado* | f/b/m (P) | M | F2,F3 | 22 |
|  | *Lutjanus jocu* | f/b/m (A) | A | F3 | 22 |
|  | *Lutjanus* sp. | f/b/m (A/P) | P | F3 | 56 |
| Megalopidae | *Megalops atlanticus* | f/b/m (A) | G | F3 | 11 |
| Mugilidae | *Dajaus monticola* | f/b/m (A/P) | C | F2 | 322 |
|  | *Mugil hospes* | b/m (A/P) | P | F2,F3 | 178 |
|  | *Mugil incilis* | f/b/m (A) | A,M | F2,F3 | 589 |
|  | *Mugil liza* | f/b/m (A) | A,G | F2,F3 | 78 |
|  | *Mugil* sp. | f/b/m (A/P) | C,M,P | F2,F3 | 1039 |
| Mullidae | *Pseudupeneus grandisquamis* | b/m (P) | P | F3 | 19 |
| Muraenidae | *Echidna catenata* | m (A) | A | F3 | 46 |
| Myliobatidae | *Rhinoptera* sp. | b/m (A/P) | P | F3 | 26 |
| Ophichthidae | *Ahlia egmontis* | b/m (A) | A | F3 | 11 |
| Poeciliidae | ***Gambusia holbrooki*** | f/b (As) | G | F3 | 59 |
|  | ***Poecilia mexicana*** | f (As) | C | F3 | 13 |
| Pomacanthidae | *Pomacanthus zonipectus* | m (P) | P | F2 | 15 |
| Pomacentridae | *Abudefduf saxatilis* | m (A) | A | F3 | 285 |
|  | *Abudefduf taurus* | m (A) | A | F3 | 13 |
|  | *Abudefduf troschelii* | b/m (P) | P | F3 | 1132 |
|  | *Stegastes* sp. | b/m (A/P) | A | F3 | 332 |
| Pristigasteridae | *Opisthopterus equatorialis* | m (P) | P | F3 | 74 |
| Scaridae | *Scarus* sp. | m (A/P) | P | F3 | 578 |
|  | *Sparisoma rubripinne* | m (A) | A | F3 | 37 |
| Sciaenidae | *Bairdiella armata* | b/m (P) | P | F3 | 33 |
|  | *Bairdiella ronchus* | f/b/m (A) | A | F3 | 224 |
|  | *Cynoscion phoxocephalus* | b/m (P) | P | F3 | 24 |
|  | *Cynoscion* sp. | f/b/m (A/P) | P | F2,F3 | 76 |
|  | *Isopisthus remifer* | b (P) | P | F3 | 49 |
|  | *Menticirrhus nasus* | b/m (P) | P | F2 | 10 |
|  | *Micropogonias* sp. | b/m (A/P) | A,G,P | F2,F3 | 221 |
|  | *Ophioscion scierus* | b/m (P) | P | F3 | 81 |
|  | *Ophioscion* sp. | b/m (A/P) | P | F2 | 388 |
|  | *Paralonchurus dumerilii* | b/m (P) | P | F3 | 123 |
|  | *Stellifer ericymba* | b/m (P) | P | F3 | 19 |
|  | *Stellifer illecebrosus* | b/m (P) | P | F3 | 225 |
|  | *Stellifer oscitans* | b/m (P) | P | F3 | 41 |
|  | *Stellifer* sp. | b/m (A/P) | P | F2,F3 | 144 |
| Scombridae | *Scomberomorus sierra* | b/m (P) | C | F3 | 28 |
| Epinephelidae | *Epinephelus itajara* | b/m (A) | A | F2 | 25 |
|  | *Epinephelus quinquefasciatus* | b/m (P) | P | F3 | 12 |
| Grammistidae | *Rypticus randalli* | b/m (A) | A | F3 | 357 |
|  | *Rypticus subbifrenatus* | m (A) | A | F3 | 234 |
| Sparidae | *Archosargus rhomboidalis* | b/m (A) | A | F3 | 316 |
| Syngnathidae | *Microphis lineatus* | f/b/m (A) | G | F3 | 311 |
| Tetraodontidae | *Arothron hispidus* | b/m (P | P | F3 | 14 |
|  | *Sphoeroides lobatus* | b/m (P) | P | F3 | 98 |
|  | *Sphoeroides spengleri* | m (A) | A | F3 | 59 |
|  | *Sphoeroides testudineus* | f/b/m (A) | P | F3 | 26 |
| **Total** | **142** |  | | | **178976** |

**Table S6** Pairwise comparisons (Tukey’s HSD) of alpha-diversity estimates for different sections of the Panama Canal. Significant results (p < 0.05) are marked in bold.

| **Section pair** | **p-value (Shannon)** | **p-value (Inverse Simpson)** |
| --- | --- | --- |
| Miraflores – Pacific | 0.125 | **0.005** |
| Culebra Cut – Pacific | **0.002** | **0.000** |
| Lake Gatun – Pacific | **0.000** | **0.000** |
| Atlantic – Pacific | 0.906 | 0.539 |
| Culebra Cut – Miraflores | 0.908 | 0.996 |
| Lake Gatun – Miraflores | 0.107 | 0.391 |
| Atlantic – Miraflores | 0.420 | 0.133 |
| Lake Gatun – Culebra Cut | 0.420 | 0.477 |
| Atlantic – Culebra Cut | **0.023** | **0.017** |
| Atlantic – Lake Gatun | **0.000** | **0.000** |
